# Annealing‐Induced Modifications in Yam Starches: Implications for Fermented Yam Flour (*Elubo*) and Textural Quality of Named Indigenous Yam Food Product—*Amala*

**DOI:** 10.1002/fsn3.72058

**Published:** 2026-06-30

**Authors:** Abiola Tanimola, Oluyinka Oroniran, Ayomide Alamu, Olawuyi Yetunde, Rahman Akinoso, Bolanle Otegbayo

**Affiliations:** ^1^ Department of Food Science and Technology, College of Agriculture, Engineering and Sciences Bowen University Iwo Osun State Nigeria; ^2^ Department of Nutrition and Dietetics, College of Health Sciences Bowen University Iwo Osun State Nigeria; ^3^ Department of Human Nutrition and Dietetics, Faculty of Public Health University of Ibadan Ibadan Oyo State Nigeria; ^4^ Department of Food Technology, Faculty of Engineering University of Ibadan Ibadan Oyo State Nigeria

**Keywords:** *amala*, *Dioscorea* species, fermented yam flour, pasting characteristics, starch annealing

## Abstract

Fermented yam flour (*elubo*) is an intermediate product for preparation of thick paste (“*amala*”) from yam tubers, commonly consumed in some parts of West Africa. Production of *elubo* involves a starch annealing process believed to influence the textural quality of the final product: *amala*. However, limited information exists on the mechanism by which the processing method of *elubo* affects the textural quality of *amala*. This study investigated the effect of the annealing process on the pasting characteristics of yam starch during the production of *elubo* and its effect on the textural quality of *amala*. Fifty‐five varieties of yam from *Dioscorea rotundata* and *Dioscorea*. *Alata* were used. Pasting characteristics of fresh yam paste and *elubo* were determined by means of Rapid Visco Analyzer. *Elubo* and *amala* samples were processed by traditional methods. Descriptive sensory evaluation by trained panelists was used to identify key food quality attributes of *amala*. Results showed that in both yam species, *elubo* had lower peak viscosity, breakdown viscosity final viscosity and setback viscosity but higher holding strength, peak time and pasting temperature compared to the fresh yam paste. Key quality attributes in *amala* were stretchability, hardness, color and stickiness. Peak, holding strength and final viscosities significantly correlated with these food quality attributes n *amala*. This study showed that yam starch annealing significantly altered the pasting characteristics of *elubo* and the textural quality of their *amala* samples. These Pasting characteristics of *elubo* (peak, holding strength, final viscosities) could serve as important determinants of food quality attributes in *amala*.

## Graphical abstract

Processing yam tubers into *elubo* (fermented yam flour) induces starch annealing, which modifies starch functionality and pasting behavior. These changes significantly influence the textural quality of *amala* prepared from 55 varieties of two *Dioscorea* species (*D. alata* and *D. rotundata*).

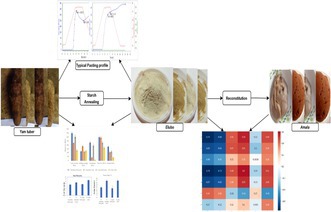

## 1 Introduction

Yam is a starchy carbohydrate crop. It is an important source of nutritional energy for the majority of people in the sub‐Saharan African region. Yams are of cultural, economic and nutritional importance because they produce edible starchy storage tubers (Otegbayo et al. 2021; Honfozo et al. 2020). There are more than 600 species of yams, out of which six are socially and economically important in terms of food, cash and medicine in West Africa. This includes *D. rotundata* (white yam), *D. alata* (water yam), *D. cayenensis* (yellow yam), *D. dumetorum* (bitter yam), *D. bulbifera* (aerial yam), and *D. esculenta* (lesser yam) (Liang et al. 2026; Obidiegwu et al. 2026; WCSP 2020).

Yam starch constitutes about 60%–80% starch of its dry matter, which is a major determinant of the characteristics of food products from yam species (Xiao et al. 2023). Yam is utilized in various forms: as boiled yam, roasted yam, pounded yam (peeled, boiled and pounded), and fermented, dried, milled and reconstituted into a thick paste called *amala*. *Amala* is a popular traditional yam food product in the West Africa region; Ghana (*Kokonte or face the wall*), Togo (*telubo*), and Republic of Benin (*telubo*). Fermented yam flour (*elubo*) is the intermediate product used for preparing *amala*, the final product. In southwestern Nigeria, *amala* has become a popular dish in many public and traditional feasting gatherings. Awoyale et al. (2020) reported that it is the second processed and acceptable yam food product after pounded yam in Nigeria.

Textural quality is one of the significant food quality attributes that influences consumer acceptability of yam food products (Ayetigbo et al. 2025; Otegbayo et al. 2024, 2021). Though pounded yam and *amala* are both popular yam food products, these products differ in their processing methods and their acceptable textural quality attributes by the consumers. Pounded yam processed by peeling, boiling, pounding, and kneading into a dough is expected to be very stretchable, smooth, and moldable (Ayetigbo et al. 2025), whereas *amala* made from fermented yam flour “*elubo*” (peeling, blanching of yam tuber in excess water at about 60°C–70°C, left in steep water for 24 h, dried and milled, and reconstituted to *amala*) is expected to be slightly stretchable, smooth, and moderately soft (Otegbayo 2020).

Annealing of yam starch is a process of physically treating starch by suspending in excess water (above 40% w/w) under mild temperature (≤ 70°C), above the glass transition temperature and below the gelatinization temperature (Yao et al. 2023; Anugerah et al. 2022). Previous authors have reported changes in the physicochemical properties of starches during annealing such as improving its crystalline nature and facilitating interactions between the starch chains, thermal stability, reduction in swelling power, reduction in viscosity and increase in pasting temperature (Yao et al. 2023; Zheng et al. 2023; Chi et al. 2019; Falade and Ayetigbo 2017). The process of producing fermented yam flour (*elubo*) simulates the starch annealing process because the yam tuber is heated (at 70°C) in excess water, below the gelatinization temperature of yam starch. During the production of *elubo* from yam tubers, where the yam starch is heated and suspended in water (similar to annealing process; Anugerah et al. 2022) a number of reactions occur, resulting in physical and functionality change. In appearance, the color of the white yam tubers changes to around creamish to deep brown, which has been associated with enzymatic browning reactions of peroxidase and polyphenol oxidase, as well as total phenol contents, which varies between and within species (Taranto et al. 2017; Otegbayo 2020; Otegbayo et al. 2021, 2025). In addition, the viscosity of the *elubo* reduced on reconstitution in hot water, compared with what is observed if unblanched or unsteeped yam flour is reconstituted. Hence, production of *elubo* through the process of blanching in excess water without allowing it to cook (but becoming flabby or rubbery) and steeping in water for a prolonged time could have functional alteration on the starch properties.

Pasting is the change in viscosity before, during and after the gelatinization, it is the sudden rise in viscosity during the heating cycle in starch which simulates the cooking process (Balet et al. 2019; Zeng et al. 2015). Pasting characteristics of yam tubers is one of the determinants of yam food textural quality (Effah‐Manu et al. 2022; Tanimola 2021; Otegbayo 2018). Therefore, since the production of *elubo* is a form of annealing process, there has been paucity of information on the relationship or mechanism of changes which take place during the production of *elubo* (intermediate product) and the textural quality of *amala* (the final product). To investigate this, 55 yam varieties from *D. rotundata* and *D. alata* were used for this study. This sample size was selected to ensure that natural variability and diversity are adequately represented, given that yam exhibits substantial diversity across genotypes and phenotypes. This study was therefore carried out to investigate the effect of annealing process on the pasting characteristics of yam starch during the processing of fermented yam flour (*elubo*) and its subsequent effect on the textural quality of yam food product *amala*.

## 2 Materials

Tubers from fifty‐five (55) varieties of *D. rotundata* (30 varieties) and *D. alata* (25 varieties) species were obtained from the yam breeding programs of the National Root Crops Research Institute (NRCRI), Umudike, Nigeria, and International Institute of Tropical Agriculture (IITA), Ibadan.

### 2.1 Methodology

#### 2.1.1 Fermented Yam Flour (*Elubo*) Processing

**FIGURE 1:**
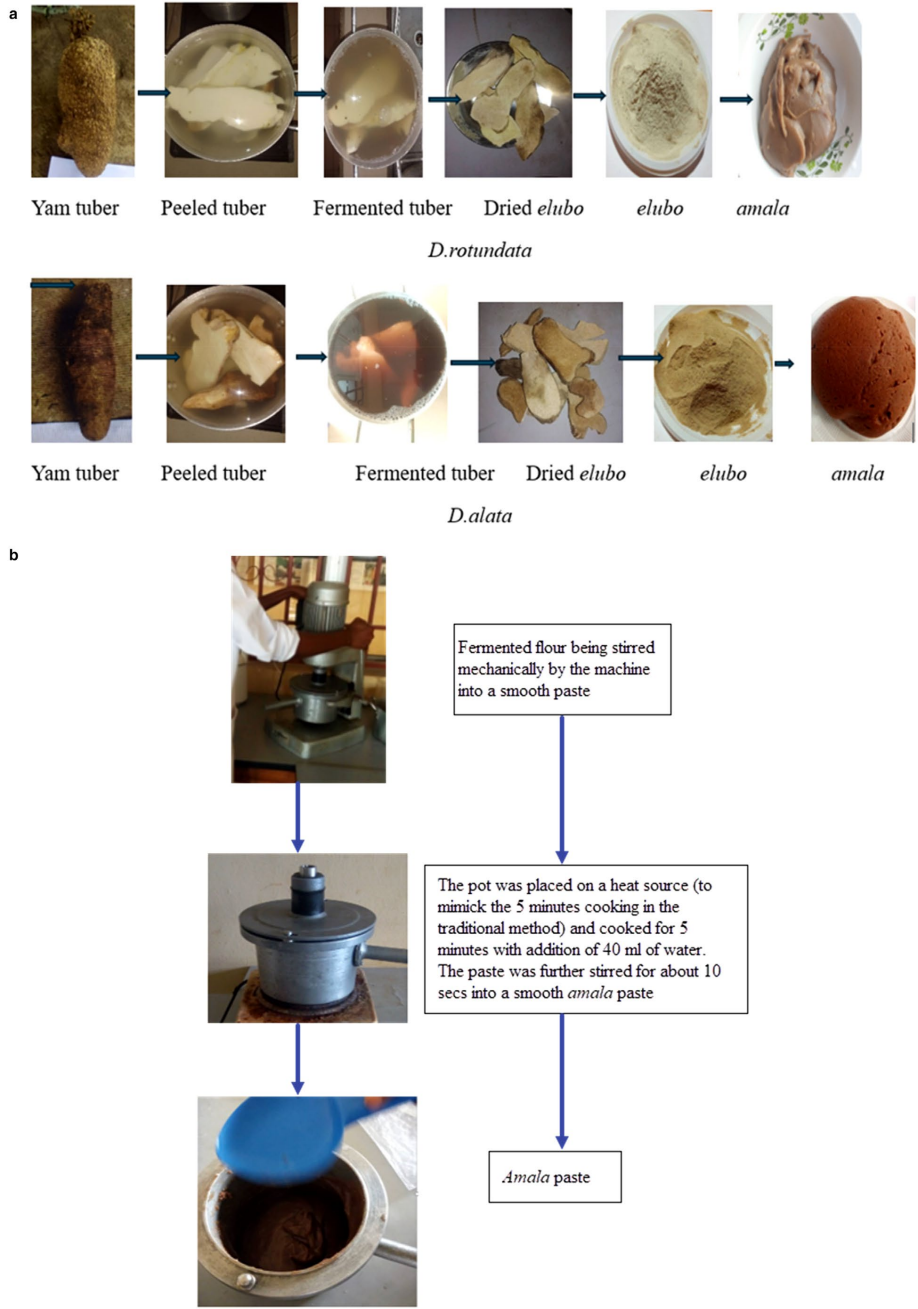
(a) Pictorial flow diagram of fermented flour (*elubo*) processing. (b) Pictorial flow diagram of preparation of *amala*.

*Elubo* was prepared from raw yam tubers by conventional method, documented by Tanimola (2021). This involved peeling of yam tubers, chipping to a size of about 15 mm by 80 mm and then blanching in submerged water on a heat source till the temperature reached 70°C (at this temperature, the yam tubers did not cook; it is below the gelatinization temperature; this is achieved when the tubers become flabby and do not break). The temperature is checked locally by putting 1–2 drops of the hot water at the back of the palm to check the degree of hotness. Appropriateness of the blanched yam piece for *elubo* was checked by taking a piece and attempting to break it; if it breaks easily then it is not ready but if it does not break (flabby or rubbery) then it is ready. The yam was left in the blanching water and steeped for 24 h, after which it was drained and sun dried until the inner core of the flabby yam was dried. The resulting dried chips were finely ground into flour (known as *elubo*) using a Panasonic grinder (MX‐AC210S, Japan) and sieved to pass through a mesh of 250 μm and (Figure 1) packaged in zip lock bags (about 3 mils thick) and stored in a desiccator, maintained at around 25°C ± 2°C, prior to reconstitution into “*amala*” and other analyses.

#### 2.1.2 Preparation of *Amala*

The conventional method of *amala* preparation (Awoyale et al. 2020) was adopted with slight modifications to ensure consistency and reproducibility. Two hundred grams of fermented yam flour (Figure 1a,b) was stirred into 500 mL of boiling water in the metal bowl pot of a stirring machine (fabricated by Addis engineering company, Nigeria). This was stirred mechanically by the machine into a smooth paste. The stirring was stopped intermittently after 10 s (this was done three times to simulate the stirring of the “*amala*” paste with pestle during traditional cooking). The pot was placed on a heat source (to mimic the 5 min cooking in the traditional method) and cooked on low heat for 5 min with the addition of 40 mL of water. The paste was further stirred for about 10 s into a smooth *amala* paste with the same consistency as the traditionally prepared *amala*. The *amala* was molded into balls and wrapped in aluminum foil and kept in a Styrofoam box to maintain the temperature (40°C) prior to sensory evaluation.

#### 2.1.3 Sensory Evaluation of “*Amala*”

Descriptive sensory evaluation method was used to evaluate the “*amala*” samples. This involved the use of extensively trained panelists to conduct the evaluation. Sixteen panelists comprising staff of Bowen University who were conversant with eating “*amala*” were used as panelist. They were selected on the basis of interest and availability and were trained on descriptive evaluation of the food products by adapting the method of Otegbayo, Aina, Sakyi‐Dawson, et al. (2005) and Otegbayo et al. (2024). Three training sessions were conducted prior to sensory evaluation. The first training session involved presentation of the objectives of the project to panelists and discussion on the food quality attributes in “*amala*.” At the 2nd training session important quality attributes in “*amala*” such as stretchability, stickiness, smoothness, color, hardness, and aroma were identified by the panelists. The panelists were given local food descriptors which exemplified the food quality attributes that were identified by the panelists (e.g., the texture of boiled egg yolk was used to exemplify smoothness, “*lafun*” (a naturally stretchy local food) was used to exemplify stretchability) was discussed and consensus was reached. At the 3rd training session each panelist was given the ballot form and “*amala*” samples were evaluated by the scoring method. For smoothness, they were given anchor points of 1–3, where 1 represents high intensity and a positive attribute (+), 2 represents medium and 3 represent negative and low intensity. However, there were some attributes that the scoring was up to 6, for instance, a scale of 1–4 was used for stretchability (very stretchable, stretchable, slightly stretchable, not stretchable), stickiness (very sticky, sticky, slightly sticky, not‐sticky), and hardness; while 1–6 was used for color (dark brown, brown, light brown, gray, light gray, very light brown), smoothness (lumpy, coarse, smooth) This was repeated twice until all panelists gave scores within ±1/4 point of the mean and were reproducible. For the sensory evaluation, the *amala* samples prepared from the yam varieties were wrapped in aluminum foil, labeled randomly and presented to the panelists in duplicates (at a temperature between 35°C and 40°C) to evaluate for stretchability, stickiness, smoothness, color, hardness, and aroma. Written and verbal informed consent were gotten from the panelists before participating in this research, the participation of all the panelists was voluntary, they were not coerced.

#### 2.1.4 Pasting Properties of Fresh Yam Paste and *Elubo*

Pasting characteristics of the fresh yam paste and *elubo* were determined by means of Rapid Visco Analyzer (RVA) (Perten Instruments of Australia 2015). The analyzer (RVA4500) connected to a PC running Thermocline for Windows (TCW) version 3 software was used to evaluate the pasting properties. The amount of sample used for the analysis was calculated based on the formula in the RVA manual:

$$\text{Corrected sample weight for}\;\mathrm{RVA}\;\left(S\right)=\frac{A\times 100}{100-M}$$

$$\left(W\right)=25-\left(S-A\right)$$

where *A* = sample weight (depending on the type of sample, this is taken from the general guide on weight of sample from RVA manual); *S* = corrected sample weight for RVA; *M* = actual moisture content of the sample; *W* = volume of water used.

The weighed sample was then blended with the measured amount of distilled water (calculated from the formula that is *W*). The parameters that were evaluated include: peak viscosity, holding strength, breakdown viscosity, final viscosity, set back viscosity, peak time and pasting temperature from the pasting profile. The standard RVA profile (RVA STD 1) comprising of initial temperature set at 50°C; holding time of 1 min at 50°C; heating to 95°C for 3 min 42 s; holding at 95°C for 2 min 30 s; cooling to 50°C for 3 min 48 s; and holding at 50°C for 2 min was used (Balet et al. 2019).

### 2.2 Statistical Analysis

Descriptive statistics and inferential methods were used to evaluate data and the relationship between pasting properties and sensory attributes of annealed yam flour products. Pearson correlation analysis was conducted to determine the linear association between individual pasting properties and sensory scores, with separate matrices computed for *D. rotundata* and *D. alata* using Python's pandas and seaborn libraries. The resulting correlation coefficients were visualized using heatmaps for clarity of interpretation. To assess statistical significance, corresponding *p*‐values were computed for correlation coefficients, and significance was evaluated at a 95% confidence level (*p* < 0.05). For predictive analysis, a multivariate regression model was implemented using the Multi Output Regressor from scikit‐learn, which fits independent linear models for each sensory attribute. Model performance was evaluated using the coefficient of determination (*R* ^2^) and Root Mean Square Error (RMSE). Statistical analyses were performed using SPSS (Version 20) and Python (Version 3.11) with relevant libraries including *pandas*, *Numpy*, *matplotlib*, *seaborn*, and *scikit‐learn*.

## 3 Results and Discussion

### 3.1 Sensory Attributes of *Amala* From *D. rotundata* and *D. alata* Varieties

The result for sensory evaluation of *amala* presented in Tables 1 and 2 showed that *amala* from *D. rotundata* varieties were generally described by the trained panelists as soft, gray in color, slightly stretchable, smooth, sticky and with bland aroma though there were some *D. rotundata amala* samples that were not stretchable, slightly sticky, and hard (Ozibo, Mailakwsa, Giwa, Adaka, Sand paper), whereas those from *D. alata* varieties were described as hard, brown in color, slightly stretchable, slightly sticky, coarse (not smooth) with bland aroma. Stretchability was described as the degree to which a sample can be extended or stretched. Most *amala* from *D. rotundata* varieties were slightly stretchable, whereas few of *D. alata* varieties were slightly stretchable. In terms of hardness, *amala* from *D. rotundata* varieties were described as softer than those of *D. alata* varieties, which were described as hard (firm or rigid) by the panelists. In terms of smoothness, *amala* samples from *D.alata* were described as coarse while those from D.*rotundata* were smooth. Stickiness was defined as how the product sticks to the finger when touched, *amala* samples from both yam species were described by the panelists as slightly sticky (Tables 1 and 2).

**TABLE 1:** Sensory attributes of *amala* made from *Dioscorea alata*.

Variety^†^	Stretchability^‡^	Stickiness	Smoothness	Hardness	Aroma	Color
TDa11/00300	3.57 ± 0.05^fed^	2.87 ± 0.00^gf^	2.60 ± 0.10^ba^	2.37 ± 0.14^ihgf^	1.54 ± 0.09^edc^	2.20 ± 0.10^ed^
TDa11/00555	3.46 ± 0.00^gfe^	3.15 ± 0.00^d^	2.66 ± 0.05^ba^	2.43 ± 0.16^hgfed^	1.62 ± 0.11^cb^	2.27 ± 0.16^d^
TDa00/00194	2.36 ± 0.10^j^	1.90 ± 0.05^j^	2.68 ± 0.05^ba^	2.75 ± 0.06^cb^	1.57 ± 0.00^dc^	2.21 ± 0.00^ed^
TDa07/00015	3.54 ± 0.15^gfed^	3.11 ± 0.05^ed^	2.57 ± 0.10^ba^	2.07 ± 0.10^mlkj^	1.50 ± 0.00^fedc^	2.07 ± 0.00^ed^
Ebina	3.64 ± 0.05^fed^	2.93 ± 0.00^fe^	2.47 ± 0.09^edcb^	2.10 ± 0.14^lkj^	1.47 ± 0.00^fedc^	2.07 ± 0.00^ed^
TDa11/00203	3.20 ± 0.10^h^	2.70 ± 0.14^hg^	2.60 ± 0.10^ba^	2.20 ± 0.10^jih^	1.44 ± 0.05^fed^	2.83 ± 0.14^c^
OA49 (TDa)	3.70 ± 0.11^dcb^	3.30 ± 0.11^dcb^	2.47 ± 0.22^edcb^	2.08 ± 0.11^mlkj^	1.81 ± 0.06^a^	1.50 ± 0.06^gf^
TDa01/00003	3.70 ± 0.12^dcb^	3.25 ± 0.1^dc^	2.53 ± 0.12^dcba^	2.47 ± 0.20^gfed^	1.83 ± 0.00^a^	1.31 ± 0.04^hg^
TDa11/00204	3.35 ± 0.05^hg^	2.62 ± 0.1^h^	2.54 ± 0.11^cba^	2.04 ± 0.06^mlkj^	1.54 ± 0.00^edc^	3.27 ± 0.06^b^
TDa 291	3.72 ± 0.00^dcb^	2.89 ± 0.00^f^	2.45 ± 0.08^edcb^	2.55 ± 0.16^fedc^	1.39 ± 0.08^fe^	3.36 ± 0.04^b^
TDa 11/00302	3.86 ± 0.10^cba^	3.29 ± 0.11^dcb^	2.33 ± 0.05^edc^	2.11 ± 0.05^lkj^	1.50 ± 0.10^fedc^	3.32 ± 0.16^b^
TDa 11/00201	2.30 ± 0.04^j^	2.27 ± 0.00^i^	2.67 ± 0.09ba	3.10 ± 0.04^a^	1.34 ± 0.09^f^	3.50 ± 0.14^b^
Civeda122	3.70 ± 0.14^dcb^	3.27 ± 0.09^dc^	2.57 ± 0.05^ba^	2.64 ± 0.05^edcb^	1.57 ± 0.14^dc^	1.67 ± 0.00^f^
Civeda136	3.88 ± 0.18^cba^	3.16 ± 0.13^d^	2.75 ± 0.08^a^	2.41 ± 0.13^gfed^	1.78 ± 0.04^ba^	1.16 ± 0.04^h^
TDa11/00014	3.44 ± 0.05^gf^	2.57 ± 0.05^h^	2.50 ± 0.14^dcb^	2.80 ± 0.00^b^	1.50 ± 0.04^fedc^	3.37 ± 0.23^b^
TDa02/00012	3.57 ± 0.10^fed^	2.93 ± 0.10^fe^	2.57 ± 0.10^ba^	2.18 ± 0.16^kjlh^	1.47 ± 0.05^fedc^	1.68 ± 0.05^f^
TDa11/00024	3.97 ± 0.05^a^	3.50 ± 0.10^a^	2.25 ± 0.06^e^	2.07 ± 0.10^mlkj^	1.75 ± 0.06^ba^	2.00 ± 0.00^e^
CIVEda 140	2.94 ± 0.09^i^	2.37 ± 0.05^i^	2.63 ± 0.14^ba^	2.60 ± 0.10^fedcb^	1.54 ± 0.09^edc^	2.10 ± 0.14^ed^
CIVEda 147	3.86 ± 0.10^cba^	3.40 ± 0.15^cba^	2.47 ± 0.05^edcb^	1.90 ± 0.05^ml^	1.43 ± 0.00^fed^	2.68 ± 0.16^c^
AZA	3.36 ± 0.10^hg^	3.11 ± 0.05^ed^	2.50 ± 0.00^dcb^	2.36 ± 0.10^ihgf^	1.40 ± 0.05^fed^	2.18 ± 0.16^ed^
Angawa Agbo	3.67 ± 0.09^edc^	2.94 ± 0.09^fe^	2.30 ± 0.14^ed^	2.27 ± 0.09^jihg^	1.57 ± 0.14^dc^	3.84 ± 0.05^a^
TDa99/00240	3.87 ± 0.00^cba^	3.47 ± 0.09^ba^	2.57 ± 0.05^ba^	2.14 ± 0.09^mlkji^	1.77 ± 0.05^ba^	1.14 ± 0.09^h^
TDa11/00510	3.62 ± 0.00^fed^	2.84 ± 0.11^gf^	2.58 ± 0.06^ba^	2.66 ± 0.05^dcb^	1.46 ± 0.11^fedc^	2.12 ± 0.05^ed^
TDa11/00179	3.88 ± 0.00^ba^	3.28 ± 0.04^dcb^	2.60 ± 0.05^ba^	1.94 ± 0.08^mlk^	1.57 ± 0.09^dc^	2.19 ± 0.08^ed^
Sugar Agbo	3.67 ± 0.09^edc^	3.27 ± 0.00^dc^	2.33 ± 0.00^edc^	1.84 ± 0.05^m^	1.64 ± 0.05^cb^	2.24 ± 0.05^ed^
Mean	3.51	2.97	2.53	2.32	1.56	2.33
STDEV	0.43	0.40	0.12	0.32	0.14	0.76
SE	0.09	0.08	0.03	0.06	0.03	0.16

Abbreviations: SD, standard deviation; SE, standard error.

^†^ Very stretchable‐1, stretchable‐2, slightly stretchable‐3, not stretchable‐4: Stickiness: Very sticky‐1, sticky‐2, slightly sticky‐3, not‐sticky‐4: Smoothness: Lumpy‐1, coarse‐2, smooth‐3: Hardness: very hard‐1, hard‐2, soft‐3, very soft‐4: Aroma: Pleasant‐1, bland‐2, unpleasant‐3: Color: Dark brown‐1, brown‐2, light brown‐3, Gray‐4, light gray‐5, very light brown‐6.

^‡^ Means with same superscripts in the same column are not significant at 5% (*p* < 0.05) level of significance.

**TABLE 2:** Sensory attributes of *amala* made from *Dioscorea rotundata*.

Variety^†^	Stretchability^‡^	Stickiness	Smoothness	Hardness	Aroma	Color
TDr0900002	2.79 ± 0.13^b^	2.53 ± 0.04^b^	2.91 ± 0.04^dcba^	2.63 ± 0.09^m^	1.38 ± 0.00^ihgf^	4.63 ± 0.09^ed^
TDr 0900082	2.04 ± 0.05^hgfed^	2.24 ± 0.05^d^	2.76 ± 0.05^hgfedc^	2.94 ± 0.09^lk^	1.40 ± 0.10^ihgfe^	4.06 ± 0.09^i^
TDr1100034	2.50 ± 0.00^dcb^	2.50 ± 0.10^b^	2.75 ± 0.06^hgfed^	3.00 ± 0.00^lkj^	1.71 ± 0.11^ba^	4.33 ± 0.05^hg^
TDr1100582	2.24 ± 0.05^fedc^	2.20 ± 0.10^d^	2.90 ± 0.04^edcba^	3.07 ± 0.00^kjih^	1.44 ± 0.05^hgfedc^	3.70 ± 0.14^j^
Ejikeme TDr	1.50 ± 0.04^jih^	1.83 ± 0.14^hgfe^	2.87 ± 0.00^fedcba^	3.34 ± 0.09^ed^	1.60 ± 0.10^dcba^	4.60 ± 0.10^ed^
TDr11‐00276	1.40 ± 0.10^ji^	1.96 ± 0.05^fe^	2.94 ± 0.09^cba^	3.34 ± 0.09^ed^	1.46 ± 0.09^hgfedc^	4.20 ± 0.10^ih^
Ekpe	1.50 ± 0.04^jih^	1.80 ± 0.10^hfe^	2.83 ± 0.14^gfedcba^	3.27 ± 0.09^gfe^	1.30 ± 0.04^ih^	4.27 ± 0.09^hg^
TDr 2665	1.69 ± 0.08^jhi^	1.82 ± 0.09^hgfe^	3.00 ± 0.00^a^	3.28 ± 0.04^fed^	1.31 ± 0.00^ih^	2.63 ± 0.09^l^
TDr 0900058	1.29 ± 0.11^j^	1.46 ± 0.05^j^	2.97 ± 0.05^ba^	3.54 ± 0.05^cb^	1.57 ± 0.10^edcba^	3.83 ± 0.05^j^
TDr 00163	2.19 ± 0.06^gfedc^	2.19 ± 0.06^d^	2.77 ± 0.00^hgfedc^	2.93 ± 0.11^lk^	1.42 ± 0.06^hgfed^	5.12 ± 0.05^a^
AMOLA TDr	1.62 ± 0.00^jih^	1.77 ± 0.00^ihgf^	2.73 ± 0.06^hgfe^	3.12 ± 0.05^jihg^	1.42 ± 0.06^hgfed^	4.39 ± 0.11^hgf^
TDr1100396	2.34 ± 0.13^edcb^	2.53 ± 0.04^b^	2.82 ± 0.09^gfedcb^	3.16 ± 0.13^jihgf^	1.50 ± 0.08^gfedc^	4.94 ± 0.00^ba^
TDr 1100492	1.58 ± 0.06^jih^	1.77 ± 0.00^ihgf^	2.70 ± 0.11^hgf^	3.04 ± 0.06^kji^	1.62 ± 0.11^cba^	4.62 ± 0.11^ed^
TDr11‐00302	2.33 ± 0.05^edcb^	2.29 ± 0.11^dc^	2.86 ± 0.10^fedcba^	2.64 ± 0.10^m^	1.54 ± 0.15^fedcb^	1.11 ± 0.05^p^
TDr100107	2.59 ± 0.12^cb^	2.20 ± 0.04^d^	2.64 ± 0.04^h^	3.22 ± 0.08^hgfed^	1.44 ± 0.00^hgfedc^	3.42 ± 0.04^k^
Fakinsa TDr	1.60 ± 0.10^jih^	1.73 ± 0.09^ihg^	2.87 ± 0.00^fedcba^	3.60 ± 0.00^ba^	1.30 ± 0.04^ih^	4.64 ± 0.05^d^
SAND PAPER TDr	1.70 ± 0.12^jihg^	1.97 ± 0.04^e^	2.67 ± 0.16^hg^	3.09 ± 0.04^kjih^	1.22 ± 0.00^i^	2.03 ± 0.04^n^
Mailakwusa	3.67 ± 0.08^a^	2.97 ± 0.04^a^	2.62 ± 0.08^h^	2.34 ± 0.08^n^	1.56 ± 0.08^fedcb^	4.53 ± 0.04^fed^
Obiaturugo	2.55 ± 0.08^dcb^	2.42 ± 0.04^cb^	2.83 ± 0.00^gfedcba^	2.86 ± 0.04^l^	1.34 ± 0.08^ihg^	3.45 ± 0.08^k^
TDr ABBI	2.68 ± 0.05^cb^	2.22 ± 0.11^d^	2.83 ± 0.05^gfedcba^	2.68 ± 0.05^m^	1.57 ± 0.00^edcba^	4.43 ± 0.00^gfe^
Okpokitora	2.43 ± 0.14^dcb^	2.23 ± 0.14^d^	2.94 ± 0.09^cba^	3.20 ± 0.00^ihgfe^	1.53 ± 0.00^fedcb^	1.34 ± 0.09^o^
Hambakwasi	1.36 ± 0.04^ji^	1.64 ± 0.04^jih^	2.97 ± 0.04^ba^	3.50 ± 0.08^cb^	1.53 ± 0.12^fedc^	4.73 ± 0.08^dc^
ADAKA TDr	1.78 ± 0.08^jihgf^	1.84 ± 0.08^hgfe^	2.97 ± 0.04^ba^	3.28 ± 0.00^fed^	1.56 ± 0.00^fedcb^	2.45 ± 0.23^m^
UHSEKPE	1.85 ± 0.00^ihgfe^	1.85 ± 0.11^gfe^	2.92 ± 0.00^dcba^	3.54 ± 0.11^cb^	1.50 ± 0.06^gfedc^	4.08 ± 0.11^i^
GIWA	2.44 ± 0.00^dcb^	2.17 ± 0.08^d^	2.86 ± 0.11^fedcba^	3.09 ± 0.04^kjih^	1.75 ± 0.11^a^	1.25 ± 0.11^po^
OZIBO	3.77 ± 0.00^a^	3.08 ± 0.11^a^	2.46 ± 0.11^i^	2.58 ± 0.06^m^	1.58 ± 0.06^edcba^	3.34 ± 0.05^k^
TDr 1106873	1.47 ± 0.15^ji^	1.61 ± 0.15^ji^	2.90 ± 0.05^edcba^	3.43 ± 0.00^dc^	1.54 ± 0.05^fedcb^	4.93 ± 0.00^ba^
Mumuye	1.54 ± 0.09^jih^	1.96 ± 0.05^fe^	2.87 ± 0.00^fedcba^	3.07 ± 0.00^kjih^	1.23 ± 0.14^i^	4.56 ± 0.05^dcb^
PUNCH	1.29 ± 0.11^j^	1.67 ± 0.05^ihg^	2.93 ± 0.10^cba^	3.25 ± 0.06^gfe^	1.57 ± 0.10^edcba^	4.90 ± 0.05^cb^
OGOJA	2.19 ± 1.15^gfedc^	1.70 ± 0.11^ihg^	3.00 ± 0.00^a^	3.73 ± 0.06^a^	1.46 ± 0.00^hgfedc^	5.08 ± 0.11^ba^
Mean	2.78	2.53	2.91	3.12	1.38	4.63
STDEV	0.64	0.39	0.13	0.33	0.13	1.17
SE	0.52	0.47	0.54	0.49	0.26	0.86

Abbreviations: SD, standard deviation; SE, standard error.

^†^ Very stretchable‐1, stretchable‐2, slightly stretchable‐3, not stretchable‐4: Stickiness: Very sticky‐1, sticky‐2, slightly sticky‐3, not‐sticky‐4: Smoothness: Lumpy‐1, coarse‐2, smooth‐3: Hardness: very hard‐1, hard‐2, soft‐3, very soft‐4: Aroma: Pleasant‐1, bland‐2, unpleasant‐3: Color: Dark brown‐1, brown‐2, light brown‐3, Gray‐4, light gray‐5, very light brown‐6.

^‡^ Means with same superscripts in the same column are not significant at 5% (*p* < 0.05) level of significance.

The Principal Component Analysis (PCA) result is presented in Table 3. For *D. rotundata* , PC1 indicates that stickiness (0.5327), stretchability (0.5155), and hardness (−0.5039), were the principal factors influencing sensory differentiation among the samples. PC2 as well showed some level of variation associated with color (−0.6730) and aroma (0.6591). For *D. alata* , PC1 showed that stickiness (0.5298), stretchability (0.5174), and hardness (−0.4406) were key to determining differences in samples. PC2 also showed that smoothness (0.5743) and color (−0.6317) were also influential. Overall, stretchability, stickiness and hardness, which are texture‐related attributes showed the primary source of variation in both species, whereas secondary differentiation was shown by aroma and color. These results are similar with the findings of Awoyale et al. (2020, 2025), who also reported this sensory quality attributes in *amala* produced from yam flour and also *amala* produced from sweet potato (Tables 4 and 5).

**TABLE 3:** Principal component analysis of *amala* from *Dioscorea rotundata* and *Dioscorea alata* showing contribution of sensory parameters to total variation.

Sensory parameters	*D. rotundata* ^a^		*D. alata*	
	PC1 (18.62%)	PC2 (17.76%)	PC1 (21.11%)	PC2 (17.99%)
Stretchability	*0.5155*	0.0592	*0.5174*	−0.1114
Stickiness	*0.5327*	−0.0321	*0.5298*	−0.0217
Smoothness	−0.4099	0.3181	−0.2709	*0.5743*
Hardness	*−0.5039*	0.0834	*−0.4406*	0.2228
Aroma	0.1147	*0.6591*	0.3334	0.4568
Color	−0.1239	*−0.6730*	−0.27	*−0.6317*

^a^ Figures in italics are significant at 5% level of significance.

**TABLE 4:** Pasting characteristics of fresh *Dioscorea rotundata* paste.^†^

Variety	Peak viscosity (RVU)^‡^	Trough viscosity (RVU)	Breakdown viscosity (RVU)	Final viscosity (RVU)	Setback viscosity (RVU)	Peak time (min)	Pasting temp (°C)
TDr09000002	401.39^fgh^	114.42^abcde^	284.72^fghi^	220.25^abcd^	98.03^abcd^	4.70^efg^	82.33^bcdef^
TDr 09‐000082	345.00^bcdefg^	86.38^abcd^	258.63^efgh^	244.63^abcde^	158.25^bcdefg^	4.27^abcde^	83.20^cdef^
TDr11/00034	516.47^ij^	107.86^abcde^	408.61^kl^	280.47^bcdef^	172.61^bcdefgh^	4.47^bcdef^	81.32^bcde^
TDr11/00582	268.64^bc^	103.44^abcde^	169.47^cde^	249.78^abcde^	138.72^bcdef^	4.49^bcdef^	82.92^bcdef^
Efikeme	323.33^bcdefg^	95.36^abcdee^	227.97^defg^	252.67^abcde^	157.31^bcdefg^	4.47^bcdef^	78.82^bcd^
TDr11/00278	386.83^defgh^	117.92^abcdefg^	268.92^efgh^	312.63^cdefgh^	194.71^cdefgh^	4.50^bcdef^	82.85^bcdef^
Ekpe	459.46^hij^	75.21^a^	384.25^ijkl^	301.21^cdefg^	226.00^fghi^	5.03^ghi^	82.88^bcdef^
TDr 2665	532.17^j^	139.08^efghi^	393.08^jkl^	364.13^efghi^	225.04^fghi^	4.50^bcdef^	80.33^bcde^
TDr0900058	246.14^ab^	126.64^bcdefg^	119.50^abc^	221.75^abcd^	95.13^abc^	4.90^fgh^	88.05^f^
TDR11/00163	238.64^ab^	182.72^i^	55.92^ab^	259.54^abcde^	76.83^ab^	5.63^jk^	85.58^ef^
Amola	282.92^bcde^	100.06^abcde^	182.89^cdef^	327.04^defghi^	227.00^fghi^	4.57^cdef^	81.10^bcde^
TDr11/396	433.50^ghij^	129.31^cdefgh^	304.22^ghij^	391.58^fghi^	262.31^ghi^	4.17^abc^	76.60^b^
TDr11/00492	438.83^ghij^	115.13^abcdefg^	323.71^hijk^	341.83^defghi^	226.72^fghi^	4.70^efg^	83.23^cdef^
TDr14/01040	367.33^cdefgh^	135.22^defghi^	232.14^defgh^	345.17^defghi^	209.97^efgh^	4.57^cdef^	82.18^bcdef^
TDr11/00101	300.25^bcdef^	117.81^abcdefg^	182.47^cdef^	282.96^bcdef^	165.17^bcdefgh^	4.53^bcdef^	67.33^a^
Fakinsa	301.96^bcdef^	175.58^ghi^	126.38^abcd^	431.25^hi^	255.67^ghi^	5.37^fgh^	82.35^bcdef^
Sandpaper	144.58^a^	111.25^abcdef^	33.33^a^	132.50^a^	21.25^a^	5.80^k^	82.65^bcdef^
Malaikwusa	334.75^bcdefg^	176.29^hi^	145.42^bcd^	420.75^ghi^	224.13^fghi^	4.60^cdefg^	85.00^def^
Obiaturugo	402.08^fgh^	124.47^abcdefg^	277.61^efgh^	448.25^i^	323.78^i^	3.98^a^	79.17^bcde^
Abbi	396.64^efgh^	108.69^abcde^	287.94^fghi^	311.47^cdefgh^	202.78^cdefgh^	5.27^hij^	82.58^bcdef^
Okpokitoro	519.19^ij^	92.22^abcde^	426.97^l^	365.69^efghi^	273.47^hi^	4.62^defg^	80.80^bcde^
Hambakwasi	362.54^cdefgh^	76.96^ab^	285.58^fghi^	269.96^bcdef^	193.00^cdefgh^	4.50^bcdef^	80.95^bcde^
Adaka	369.94^cdefgh^	159.33^fghi^	210.61^cdefg^	256.47^abcde^	97.14^abcd^	4.24^abcd^	80.52^bcde^
Uhsekpe	239.97^ab^	99.97^abcde^	140.00^bcd^	238.14^abcde^	138.17^bcdef^	5.22^hi^	82.25^bcdef^
Giwa	284.69^bcdefg^	87.00^abcd^	197.69^cdefg^	291.92^bcdefg^	204.92^defgh^	4.31^abcde^	80.22^bcde^
Ozibo	353.75^bcdefg^	119.86^abcdefg^	233.89^defgh^	359.28^efghi^	239.42^fghi^	4.62^defg^	81.00^bcde^
TDr110/06873	420.78^ghi^	164.22^ghi^	256.56^efgh^	356.11^efghi^	191.89^cdefgh^	4.69^defg^	83.45^cdef^
Mumuye	279.33^bcd^	86.03^abcd^	193.31^cdef^	166.03^ab^	80.00^ab^	4.11^ab^	78.27^bc^
Punch	277.33^bcd^	84.40^abc^	192.97^cdef^	187.58^abc^	103.17^abcde^	4.37^abcde^	81.54^bcde^
Ogoja	271.08^bcd^	141.19^efghi^	129.89^abcd^	274.61^bcdef^	133.42^bcdef^	4.60^cdefg^	80.80^bcde^
Mean	349.98	118.47	231.16	296.85	177.20	4.66	81.34
SD	92.15	30.05	98.19	76.65	68.90	0.43	3.45
SE	16.82	5.49	17.93	13.99	12.58	0.08	0.63

Abbreviations: RVU, Rapid Viscosity Unit; SD, standard deviation; SE, standard error.

^†^ Results are means of triplicate analysis.

^‡^ Means with same superscripts in the same column are not significant at 5% (*p* < 0.05) level of significance.

**TABLE 5:** Pasting characteristics of fresh *Dioscorea alata* paste.^†^

Varieties	Peak viscosity (RVU)^†^	Trough viscosity (RVU)	Breakdown viscosity (RVU)	Final viscosity (RVU)	Setback viscosity (RVU)	Peak time (min)	Pasting temp (°C)
TDa11/00300	342.53^jklm^	158.53^ghi^	184.00^efg^	307.50^ij^	148.97^efghi^	5.29^jkl^	74.12^abcde^
TDa11/00555	208.72^def^	98.39^cdef^	110.33^cde^	187.14^de^	88.75^bc^	4.80^ef^	84.70^de^
TDa00/00194	279.75^ghij^	72.29^abc^	207.46^fghi^	214.38^e^	142.08^efgh^	5.03^ghi^	70.08^abcde^
TDa07/00015	270.50^fghi^	68.29^abc^	202.21^fgh^	212.83^e^	144.54^efgh^	5.34^kl^	86.40^e^
Ebina	449.83^pq^	189.31^hij^	260.53^hij^	370.14^k^	180.83^hijk^	4.89^fgh^	79.98^bcde^
Tda11/00203	373.88^lmno^	165.96^ghij^	207.92^fghi^	300.42^hij^	134.46^defg^	5.20^ijkl^	86.90^e^
OA49 (TDa)	355.22^klmn^	213.58^j^	141.64^def^	314.83^ij^	101.25^bcd^	4.89^fgh^	79.85^bcde^
TDa01/00003	329.67^ijkl^	64.36^abc^	265.31^hij^	222.69^ef^	158.33^fghij^	4.42^b^	83.52^bcde^
TDa11/00204	185.54^cde^	128.33^defg^	57.21^abc^	165.42^d^	37.08a	5.30^kl^	78.28^bcde^
TDa291	406.31^mnop^	145.56^fgh^	260.75^hij^	295.17^ghij^	149.61^efghi^	4.89^fg^	83.22^bcde^
TDa11/00302	200.69^cd^	34.35^a^	166.38d^ef^	34.15^a^	5.08a	4.07^a^	58.70^abc^
TDa11/00201	401.54^lmno^	193.00^ij^	201.00^efg^	272.17^ghi^	74.58b	4.92^fgh^	84.28^cde^
Civeda122	339.19^jklm^	85.11^bcd^	254.08^ghij^	259.33^fg^	174.22^ghij^	4.82^efg^	74.10^abcde^
Civeda136	186.33^cde^	66.75^abc^	119.58^cde^	155.28^cd^	88.53bc	5.09^hij^	85.67^de^
TDa11/00014	342.29^jkl^	73.71^abc^	268.58^hij^	191.33^de^	117.63^cde^	4.53^bc^	83.18^bcde^
TDa02/00012	475.04^q^	128.58^defg^	346.46^k^	396.08^k^	267.50^l^	4.67^cde^	84.15^bcde^
TDa11/00024	90.78^ab^	68.28^abc^	22.50^ab^	74.78^b^	6.50^a^	5.22^ijkl^	86.17^e^
Civeda 140	432.03^opq^	107.56^cdef^	324.47^jk^	293.67^ghij^	186.11^ijk^	4.87^efg^	85.08^de^
Civeda 147	414.47^nopq^	105.50^cdef^	308.97^jk^	322.47^j^	216.97^k^	4.77^ef^	84.00^bcde^
AZA	245.86^efgh^	165.67^ghij^	80.19^abcd^	191.06^de^	25.39^k^	4.75^def^	84.78^de^
Angawa Agbo	299.25^hijk^	195.31^ij^	103.97^cd^	272.89^ghij^	77.58^a^	4.57^bcd^	83.20^bcde^
TDa99/00240	229.14^defg^	140.94^fgh^	88.19^bcd^	178.69^de^	37.75b	4.80^ef^	84.98^de^
TDa11/00510	369.83^lmno^	84.25^bcd^	285.58^jk^	215.42^e^	131.17^a^	4.43^b^	82.73^bcde^
TDa11/00179	392.69^lmnop^	73.06^abc^	319.64^jk^	269.28^gi^	196.22^jk^	4.93^fgh^	85.30^de^
Sugar Agbo	52.96^a^	40.58^ab^	12.38^a^	47.63^ab^	7.04^a^	5.04^ghi^	66.75^bc^
mean	306.96	114.69	191.97	230.59	115.93	4.86	80.80
SD	109.97	52.30	98.04	91.74	70.65	0.31	6.98
SE	21.99	10.46	19.61	18.35	14.13	0.06	1.40

Abbreviations: RVU, Rapid Viscosity Unit; SD, standard deviation; SE, standard error.

^†^ Results are means of triplicate analysis.

^‡^ Means with same superscripts in the same column are not significant at 5% (*p* < 0.05) level of significance.

### 3.2 Pasting Characteristics of Fresh Yam Paste

**FIGURE 2:**
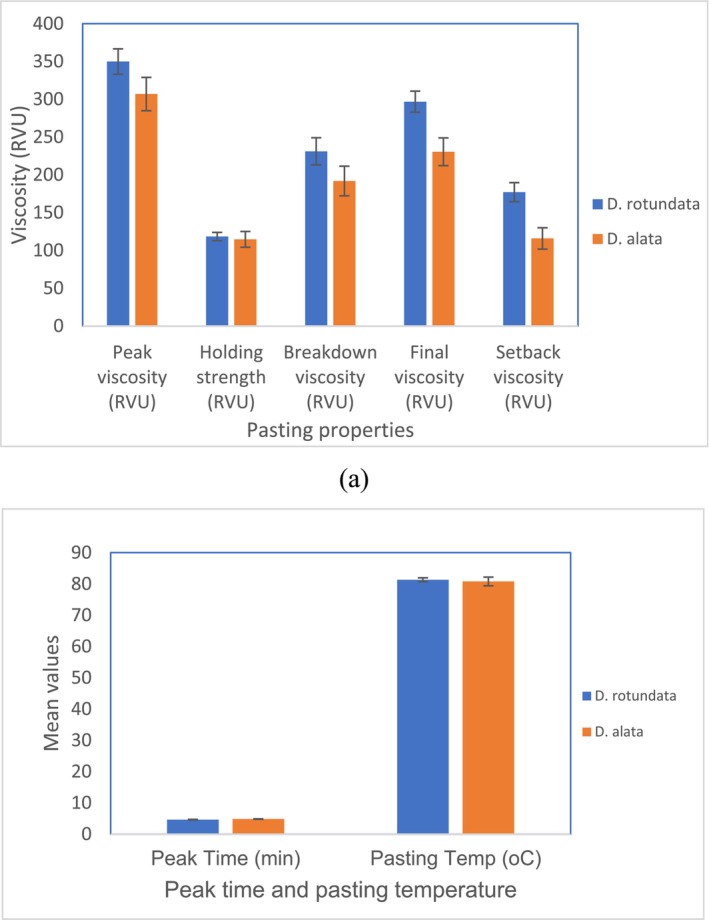
Summary of pasting characteristics of fresh yam tubers from *Dioscorea alata* and *Dioscorea rotundata*. (a) Summary of viscosities (RVU). (b) Summary of peak time (min) and pasting temperature (°C).

The result of the pasting characteristics of the different varieties of fresh yam tubers from the yam species are presented in Tables 4 and 5. The results agreed with that of Tanimola et al. (2022) and Otegbayo et al. (2006). There were significant differences (*p* < 0.05) among the pasting characteristics of the fresh yam paste from the two yam species (Figure 2a,b). Many authors have reported the multivariablity of *Dioscorea species* as reflected by the physicochemical properties, including pasting properties of *D. rotundata* and *D. alata* starches (Mawoneke et al. 2025; Tanimola et al. 2022; Otegbayo et al. 2014; Wireko‐Manu et al. 2011). *D. rotundata* had significantly (*p* < 0.05) higher mean values of peak viscosity (349.98 RVU), breakdown viscosity (231.16 RVU), final viscosity (296.85 RVU) as well as setback viscosity (177.20 RVU) compared with *D. alata* tubers. Pasting is the combined effect of swelling and rate of disruption of the granules (Batey 2007). Pasting temperature is the temperature at which the viscosity of the paste begins to increase, it is the minimum temperature required to cook a sample (Balet et al. 2019). There was no significant difference in the pasting temperature of yam starch from both species, this implied that their cooking time is about the same. Peak viscosity (PV) of starch is the highest viscosity reached during the heating and pasting period. PV signifies their water binding capacity, and the thickness (viscosity) of the starch paste after cooking (Balet et al. 2019). It occurs at the equilibrium point between granule swelling and polymer leaching which causes an increase in viscosity and granule rupture and polymer alignment due to mechanical shear which causes a decrease in viscosity. High pasting temperature and high peak viscosity has also been attributed to removal of water from the exuded amylose by the granules as they swell (Balet et al. 2019). High pasting temperature and high peak viscosity of the *D. rotundata* starches indicate that they are highly resistance to swelling and rupturing, hence they exhibited restricted swelling. Thus. *D. rotundata* varieties with higher peak viscosities will form thicker pastes on cooking. The lower breakdown and holding strength of *D. alata* fresh tubers compared to *D. rotundata* indicates lower paste stability during the hold period, thus a lower resistance to mechanical fragmentation during heating and shearing stress. As the heating of the starch progressed there was reduction in viscosity of the paste as a result of crystalline melting which allows more water into the granule, collision of the granules which led to mechanical fragmentation. This viscosity is the Breakdown viscosity (BV). The lower breakdown of *D. alata* may suggest a weak cross‐linking among its starch granules. The higher breakdown of *D. rotundata* starch indicates that there was less granule rupture for the starches, hence they have better paste stability. In the cooling stage, the viscosity of the starch pastes increases again due to retrogradation and attempt by the amylose and amylopectin of starch molecules to re‐associate and form a crystalline structure; this is called the setback viscosity (Balet et al. 2019). The higher the setback viscosity in starches the higher is the tendency of the starch to retrograde and vice‐versa (Yao et al. 2023; Sandhu and Singh 2007; Kaur et al. 2007). Thus, higher setback values observed in *D. rotundata* fresh paste, may imply that the starch will form a more cohesive paste but with higher retrogradation tendency than *D. alata* (Effah‐Manu et al. 2022; Otegbayo et al. 2014, 2006; Baah et al. 2009). Final viscosity is the ability of the material to form a viscous paste or gel after cooking and cooling. During the cooling stage, there was increase in the viscosity because the constituents of the hot paste (swollen granules, fragments of swollen granules, colloidal and molecularly dispersed starch molecules) associate as the temperature decreased. High final viscosity of the *D. rotundata* indicated that the paste is more resistant to mechanical shear and suggests the formation of a more structured paste. The implication of this result on the quality of the yam food product is that *D. rotundata* with a higher final viscosity formed a firmer gel. The exudation of starch during polymer leaching may be responsible for a more sticky product (higher stickiness/adhesiveness of the *amala* to fingers). However, there were intra specie variations among the yam varieties in terms of these pasting parameters in both *D. alata* and *D. rotundata* . Generally, viscosity differences among starches can be attributed to amylose/amylopectin ratio, granule size, arrangement in chain length distribution, amylose and protein or lipid and amylopectin structure (Balet et al. 2019).

### 3.3 Pasting Characteristics of “*Elubo*”

**FIGURE 3:**
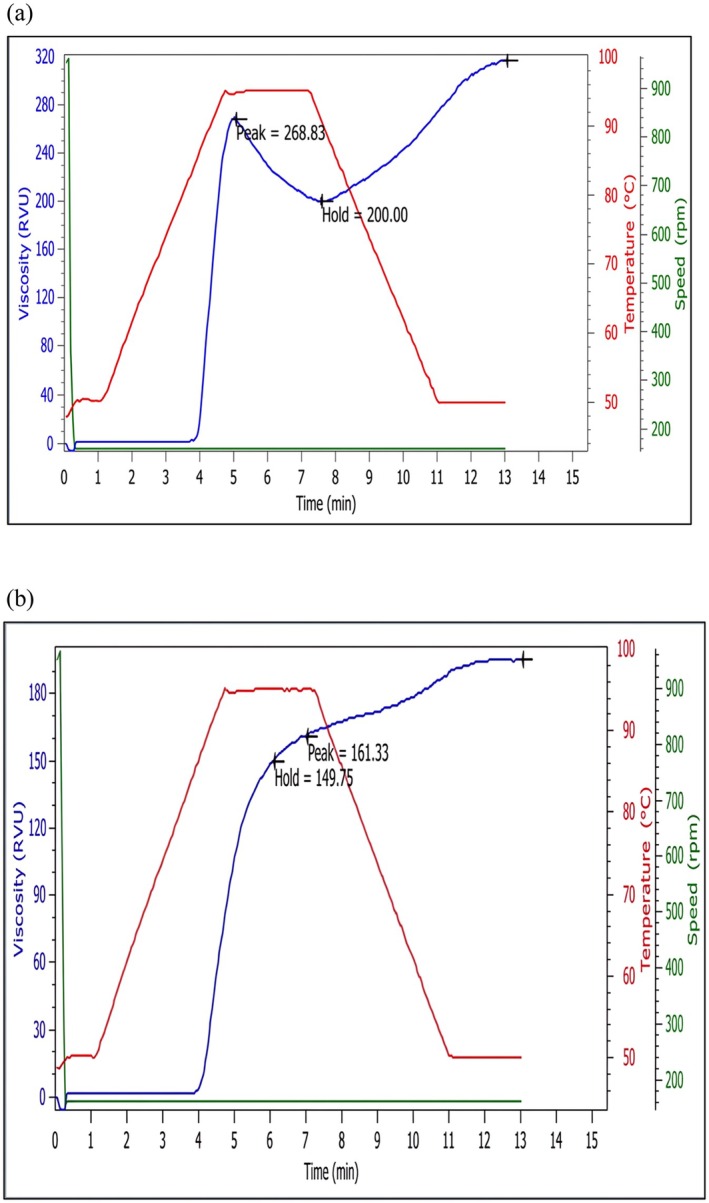
Typical pasting profile of “*elubo*” from: (a) *Dioscorea rotundata* (b) *Dioscorea alata* species.

**FIGURE 4:**
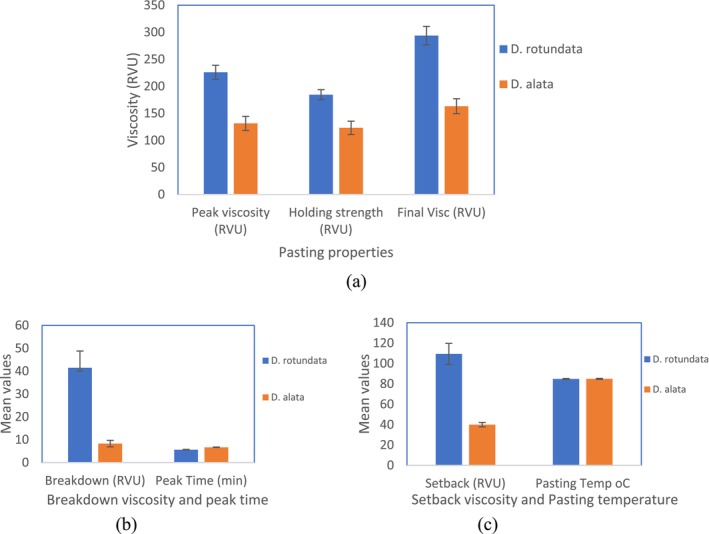
Summary of pasting characteristics of fermented yam flour “*elubo*” from *Dioscorea rotundata* and *Dioscorea alata*. (a) Summary of peak viscosity (RVU), holding strength (RVU) and final viscosity (RVU). (b) Summary of breakdown viscosity (RVU) and peak time (min). (c) Summary of setback viscosity (RVU) and pasting temperature (°C).

**FIGURE 5:**
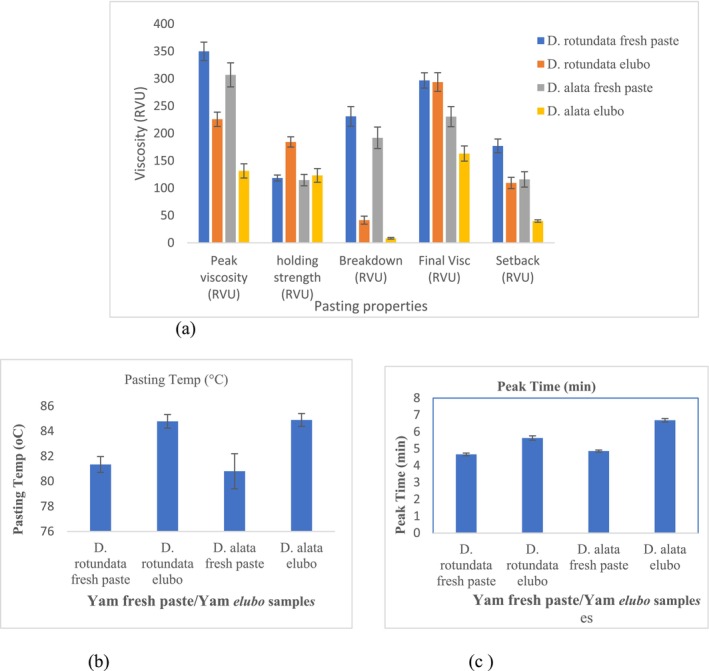
Differences between the pasting characteristics of fresh yam paste and “*elubo*.” (a) Differences between the pasting characteristics (viscosities in RVU) of fresh yam paste and “*elubo*.” (b) Differences between the pasting temperature (°C) of fresh yam paste and “*elubo*”. (c) Differences between the Peak time (min) of fresh yam paste and “*elubo*.”

The pasting characteristics of the different varieties of *elubo* prepared from the same set of fresh yam tubers described previously are presented in Tables 6 and 7. *Elubo* is the intermediate product between fresh yam tubers and *amala* (the final product). A generalized pasting profile of fermented yam flour (*elubo*) from both yam species is presented in Figure 3a,b. There were significant differences among the pasting characteristics of the *elubo* from the two yam species (Figure 4) as was observed in the fresh yam pastes (Figure 2). *Elubo* from *D. rotundata* had significantly (*p* < 0.05) higher peak viscosity (225.91 RVU), holding strength (184.44 RVU), breakdown (41.47 RVU), setback (109.42 RVU), as well as final viscosity (293.86 RVU) than *elubo* from *D. alata* varieties. However, there was no significant difference among them (*D. alata and D. rotundata*) in terms of peak time and pasting temperature (Figure 4). Generally, the pasting characteristics of *elubo* were different from the pasting characteristics of the yam starches/fresh yam pastes (Figure 5a–c). This may be attributed to physical modification or annealing of the starch which occurred during the process of blanching and steeping for 24 h, and the fermentation process which took place during the production of *elubo* from the yam tubers.

**TABLE 6:** Pasting characteristics of “elubo” from *Dioscorea rotundata*.^†^

Variety	Peak viscosity (RVU)^‡^	Holding strength * viscosity (RVU)	Breakdown viscosity (RVU)	Final viscosity (RVU)	Setback viscosity (RVU)	Peak time (min)	Pasting temp (°C)
TDr0900002	260.95 ± 5.38^b^	255.64 ± 5.48^b^	5.31 ± 0.84^dcba^	341.39 ± 7.22^m^	85.75 ± 2.21^ihgf^	5.58 ± 0.14^ed^	89.97 ± 0.42hgf
TDr 0900082	200.53 ± 5.74h^gfed^	171.05 ± 4.56^d^	29.47 ± 1.22^hgfedc^	244.00 ± 6.06^lk^	72.95 ± 1.77^ihgfe^	4.95 ± 0.04^i^	88.83 ± 0.08 dc
TDr1100034	268.58 ± 3.72^dcb^	176.08 ± 13.98^b^	92.50 ± 10.38^hgfed^	294.83 ± 3.94^lk^j	118.75 ± 10.10^ba^	4.82 ± 0.04^hg^	86.22 ± 0.49 dc
TDr1100582	268.94 ± 2.17^fedc^	206.83 ± 7.59^d^	62.11 ± 6.37^edcba^	318.33 ± 4.06^kjih^	111.50 ± 5.04^hgfedc^	5.05 ± 0.04^j^	87.62 ± 0.39 kjih
Ejikeme TDr	270.28 ± 7.54^jih^	225.42 ± 3.69^hgfe^	44.86 ± 10.78^fedcba^	373.09 ± 6.87^ed^	147.67 ± 10.20^dcba^	5.27 ± 0.07^ed^	85.88 ± 0.49hgf
TDr11‐00276	312.92 ± 2.45^ji^	257.47 ± 4.84^fe^	55.45 ± 4.22^cba^	408.47 ± 6.04^ed^	151.00 ± 6.47^hgfedc^	5.24 ± 0.25^ih^	87.77 ± 0.42hgf
Ekpe	262.36 ± 2.75^jih^	175.53 ± 30.33^hgfe^	86.83 ± 30.83^gfedcba^	327.86 ± 9.33^gfe^	152.33 ± 39.15^ih^	4.95 ± 0.04^hg^	85.90 ± 0.48kj
TDr 2665	207.75 ± 3.40^jihg^	194.47 ± 4.03^hgfe^	13.28 ± 0.67^a^	258.61 ± 3.42^fed^	64.14 ± 1.49^ih^	7.00 ± 0.00^l^	82.25 ± 0.05 m
TDr 0900058	290.22 ± 4.80^j^	219.00 ± 16.82^j^	71.22 ± 13.02^ba^	358.11 ± 4.82^cb^	145.56 ± 10.29^edcba^	4.98 ± 0.08^j^	88.10 ± 0.05fe
TDr 00163	274.03 ± 2.43^gfedc^	217.11 ± 2.51^d^	56.92 ± 0.09^hgfedc^	324.33 ± 2.55^lk^	107.22 ± 1.44^ihgfed^	4.96 ± 0.08^a^	87.33 ± 0.03fe
AMOLA TDr	234.86 ± 1.92^jih^	198.78 ± 12.08^ihgf^	36.08 ± 11.91^hgfe^	300.53 ± 4.94^jihg^	101.75 ± 7.23^hgfed^	5.35 ± 0.17^hgf^	87.75 ± 0.48lk
TDr1100396	172.67 ± 8.47^edcb^	170.89 ± 8.27^b^	1.78 ± 0.27^gfedcb^	228.33 ± 9.96^ihgf^	57.45 ± 2.00^gfed^	5.60 ± 0.12^ba^	82.60 ± 0.56kjihg
TDr 1100492	234.30 ± 3.39^jih^	227.86 ± 3.58^ihgf^	6.44 ± 0.83^hgf^	336.94 ± 0.51^kji^	109.08 ± 3.11^cba^	6.98 ± 0.04^ed^	84.25 ± 0.48^gf^
TDr11‐00302	158.39 ± 22.71^edcb^	148.50 ± 19.06^dc^	9.89 ± 3.90^fedcba^	187.89 ± 12.17^m^	39.39 ± 6.98^fedcb^	6.67 ± 0.37^p^	82.88 ± 0.46^edc^
TDr100107	250.86 ± 23.18^cb^	196.28 ± 9.80^d^	54.58 ± 13.80^h^	301.69 ± 26.71^hgfe^	105.42 ± 17.46^hgfedc^	4.91 ± 0.08^k^	88.58 ± 0.42^jihgf^
Fakinsa TDr	278.36 ± 5.19^jih^	231.17 ± 11.49^ihg^	47.19 ± 9.84^fedcba^	376.17 ± 7.75^ba^	145.00 ± 6.83^ih^	5.33 ± 0.07^d^	85.27 ± 0.45^ihgf^
SAND PAPER TDr	230.97 ± 4.47^jihg^	200.92 ± 4.33^e^	30.05 ± 3.52^hg^	312.33 ± 6.92^kjih^	111.42 ± 7.66^i^	5.58 ± 0.10^n^	80.45 ± 0.43^ml^
Mailakwusa	103.50 ± 1.38^a^	95.39 ± 1.97^a^	8.11 ± 2.79^h^	150.08 ± 5.81^n^	54.69 ± 5.31^fedcb^	6.49 ± 0.32^fed^	82.30 ± 0.05^b^
Obiaturugo	118.50 ± 0.44^dcb^	117.36 ± 0.43^cb^	1.14 ± 0.05^gfedcba^	157.61 ± 2.22^l^	40.25 ± 1.80^ihg^	6.78 ± 0.04^k^	79.82 ± 0.83^k^
TDr ABBI	163.42 ± 3.85^cb^	143.19 ± 3.27^d^	20.22 ± 0.96^gfedcba^	218.47 ± 6.61^m^	75.28 ± 4.54^edcba^	5.47 ± 0.20^gfe^	81.00 ± 0.43^dc^
Okpokitora	144.53 ± 0.80^dcb^	135.50 ± 3.75^d^	9.03 ± 3.01^cba^	189.44 ± 1.55^ihgfe^	53.94 ± 2.41^fedcb^	5.65 ± 0.04^o^	80.98 ± 0.49^kjih^
Hambakwasi	278.55 ± 6.30^ji^	197.55 ± 24.84^jih^	81.00 ± 20.50^ba^	354.53 ± 9.83^cb^	156.97 ± 32.57^fedc^	5.11 ± 0.03^dc^	85.65 ± 0.05^lk^
ADAKA TDr	208.86 ± 15.30^jihgf^	189.78 ± 13.34^hgfe^	19.08 ± 2.17^ba^	316.83 ± 33.70^fed^	127.06 ± 20.42^fedcb^	5.51 ± 0.08^m^	80.63 ± 0.06^kjihg^
UHSEKPE TDr	174.47 ± 5.99^ihgfed^	161.30 ± 16.35^gfe^	13.17 ± 10.61^dcba^	225.81 ± 8.29^cb^	64.50 ± 8.05^gfedc^	6.04 ± 0.38^i^	82.63 ± 0.49^kji^
GIWA TDr	124.00 ± 2.98^dcb^	114.58 ± 3.23^d^	9.42 ± 0.30^fedcba^	167.00 ± 3.90^kjih^	52.41 ± 0.88^a^	7.00 ± 0.00^po^	79.88 ± 0.10^a^
OZIBO TDr	39.33 ± 1.50^a^	34.81 ± 1.33^a^	4.53 ± 0.29^i^	57.53 ± 1.97^m^	22.72 ± 0.65^edcba^	7.00 ± 0.00^k^	79.85 ± 0.00^b^
TDr 1106873	301.97 ± 5.07^ji^	271.25 ± 13.34^ji^	30.72 ± 9.86^edcba^	386.67 ± 9.14^dc^	115.42 ± 10.62^fedcb^	5.51 ± 0.10^ba^	87.80 ± 0.39^c^
Mumuye	298.14 ± 10.69^jih^	234.39 ± 31.66^fe^	63.75 ± 24.50^fedcba^	409.83 ± 28.41^kjih^	175.44 ± 56.59^i^	5.38 ± 0.33^fed^	84.33 ± 0.45^fed^
PUNCH	362.36 ± 18.67^j^	193.05 ± 45.74^ihg^	169.31 ± 27.44^cba^	455.75 ± 7.22^gfe^	262.70 ± 38.91^edcba^	4.80 ± 0.00^cb^	84.30 ± 0.44^kjih^
OGOJA	277.25 ± 4.77^gfedc^	240.53 ± 11.19^ihg^	36.72 ± 10.66^a^	410.70 ± 4.13^a^	170.17 ± 9.61^hgfedc^	5.33 ± 0.07^ba^	85.87 ± 0.55^fed^
Mean	225.91	184.44	41.47	293.86	109.42	5.63	84.78
SD	71.54	51.22	40.17	93.54	56.48	0.74	2.94
SE	13.06	9.35	7.33	17.08	10.31	0.13	0.54

Abbreviations: RVU, Rapid Viscosity Unit; SD, standard deviation; SE, standard error.

^†^ Results are means of triplicate analysis.

^‡^ Means with same superscripts in the same column are not significant at 5% (*p* < 0.05) level of significance.

**TABLE 7:** Pasting characteristics of “elubo” from *Dioscorea alata*.^†^

Variety	Peak viscosity (RVU)^‡^	Holding strength * viscosity (RVU)	Breakdown viscosity (RVU)	Final viscosity (RVU)	Setback viscosity (RVU)	Peak time (min)	Pasting temp (°C)
TDa11/00300	187.67 ± 2.22^d^	183.58 ± 3.34^d^	4.08 ± 2.10^gf^	230.53 ± 5.15^c^	46.94 ± 3.62^dc^	6.95 ± 0.04^a^	84.25 ± 0.48^ihgf^
TDa11/00555	162.14 ± 7.39^f^	160.69 ± 8.00^f^	1.45 ± 0.62^g^	204.64 ± 8.12^e^	43.94 ± 1.07^fed^	6.62 ± 0.30^ba^	87.23 ± 0.03^edcb^
TDa00/00194	245.33 ± 7.37^a^	242.89 ± 7.44^a^	2.44 ± 0.27^g^	287.19 ± 8.54^a^	44.31 ± 1.71^fedc^	6.73 ± 0.18^a^	90.43 ± 2.18^a^
TDa11‐00138	116.89 ± 3.58^i^	112.47 ± 3.94^k^	4.42 ± 0.42^gf^	151.33 ± 3.59^j^	38.86 ± 0.81^f^	7.00 ± 0.00^a^	83.17 ± 0.03^jih^
TDa07/00015	162.75 ± 3.77^f^	156.75 ± 4.99^hgf^	6.00 ± 1.26^gfed^	197.64 ± 1.81^gfe^	40.89 ± 3.23^fe^	6.98 ± 0.04^a^	86.37 ± 2.93^gfedcb^
Ebina	143.92 ± 6.81^h^	132.97 ± 7.87^j^	10.94 ± 1.09^edc^	172.36 ± 6.30^i^	39.39 ± 1.59^f^	7.00 ± 0.00^a^	83.67 ± 0.49^ihg^
TDa11‐00203	166.50 ± 4.78^f^	153.95 ± 4.48^ihgf^	12.55 ± 1.28^c^	200.83 ± 5.79^fe^	46.89 ± 1.44^dc^	7.00 ± 0.00^a^	88.45 ± 5.38^cba^
OA49 (TDa)	55.89 ± 2.11^m^	50.22 ± 2.04^n^	5.67 ± 0.22^gfe^	93.17 ± 3.12^n^	42.94 ± 1.21^fed^	7.00 ± 0.00^a^	83.95 ± 0.00^ihgf^
TDa01‐00003	20.75 ± 0.22^o^	18.25 ± 0.37^p^	2.50 ± 0.17^g^	36.28 ± 0.78^p^	18.03 ± 1.06^j^	7.00 ± 0.00^a^	80.63 ± 0.13^k^j
TDa11/00204	199.11 ± 3.69^c^	169.11 ± 3.93^e^	30.00 ± 4.46^a^	216.55 ± 3.81^d^	47.44 ± 4.55^dc^	5.42 ± 0.10^e^	88.87 ± 0.03^ba^
TDa 291	178.69 ± 3.16^e^	151.92 ± 5.15^ihg^	26.78 ± 7.49^a^	198.06 ± 2.50^gfe^	46.14 ± 5.03^edc^	5.31 ± 0.10^e^	86.73 ± 0.41^fedcb^
TDa11‐00302	165.33 ± 7.43^f^	160.33 ± 5.79^f^	5.00 ± 3.18^gfe^	190.56 ± 7.63^hg^	30.22 ± 2.74^ihg^	6.98 ± 0.04^a^	84.75 ± 2.16^ihgfe^
TDa11‐00201	223.89 ± 3.84^b^	209.00 ± 4.34^b^	14.89 ± 7.92^c^	264.92 ± 5.28^b^	55.92 ± 9.19^b^	6.11 ± 0.50^dc^	85.93 ± 0.49^hgfed^
Civeda 122	36.50 ± 1.08^n^	31.20 ± 1.28^o^	5.31 ± 0.20^gfe^	63.81 ± 0.42^o^	32.61 ± 0.92^g^	7.00 ± 0.00^a^	84.77 ± 0.08^ihgfe^
Civeda 136	22.75 ± 0.74^o^	20.83 ± 1.00^p^	1.92 ± 0.36^g^	39.58 ± 0.92^p^	18.75 ± 0.96^j^	7.00 ± 0.00^a^	79.28 ± 0.40^k^
TDa11‐00014	161.19 ± 3.51^f^	158.67 ± 1.96^gf^	2.53 ± 2.14^g^	185.42 ± 3.03^h^	26.75 ± 1.15^ih^	6.35 ± 0.71^cb^	84.27 ± 0.46^ihgf^
TDa 02‐00012	112.80 ± 2.04^i^	107.89 ± 1.74^k^	4.92 ± 0.30^gfe^	158.11 ± 2.48^j^	50.22 ± 1.05^c^	7.00 ± 0.00^a^	84.77 ± 0.03^ihgfe^
TDa11‐00022	103.56 ± 1.54^j^	93.72 ± 1.59^l^	9.83 ± 0.09^fedc^	141.28 ± 1.92^k^	47.56 ± 0.59^dc^	7.00 ± 0.00^a^	84.73 ± 0.08^ihgfe^
Civeda 140	139.64 ± 5.18^h^	127.39 ± 4.92^j^	12.25 ± 6.53^c^	174.47 ± 6.59^i^	47.08 ± 7.94^dc^	6.85 ± 0.17^a^	83.43 ± 0.45^ih^
Civeda 147	82.44 ± 2.20^l^	77.47 ± 2.33^m^	4.97 ± 0.25^gfe^	102.83 ± 0.96^m^	25.36 ± 1.38^i^	7.00 ± 0.00^a^	83.17 ± 0.03^jih^
TDa AZA	222.00 ± 4.89^b^	200.72 ± 8.16^c^	21.28 ± 5.29^b^	264.92 ± 8.28^b^	64.19 ± 2.72^a^	5.78 ± 0.10^d^	88.10 ± 0.00^dcba^
Angawa Agbo—	159.72 ± 0.63^f^	148.05 ± 4.38^ih^	11.67 ± 4.96^dc^	195.56 ± 3.15^gf^	47.50 ± 1.38^dc^	6.31 ± 0.10^cb^	85.60 ± 2.12^hgfed^
TDa 99‐00240	31.75 ± 1.01^n^	28.11 ± 1.03^o^	3.64 ± 0.10^g^	58.61 ± 0.55^o^	30.50 ± 0.59^ihg^	7.00 ± 0.00^a^	83.98 ± 0.06^ihgf^
TDa11/00510	80.61 ± 1.03^l^	74.72 ± 1.07^m^	5.89 ± 0.13^gfed^	106.14 ± 0.49^m^	31.42 ± 1.48^hg^	7.00 ± 0.00^a^	84.78 ± 0.03^ihgfe^
TDa11/00179	97.00 ± 0.83^k^	91.03 ± 0.70^l^	5.97 ± 0.38^gfed^	130.11 ± 0.13^l^	39.09 ± 0.80^f^	7.00 ± 0.00^a^	85.00 ± 0.35^ihgfe^
Sugar Agbo	152.14 ± 4.53^g^	150.00 ± 2.82^i^	2.14 ± 1.72^g^	192.14 ± 1.77^hg^	42.14 ± 2.14^fed^	5.89 ± 0.23^d^	82.32 ± 0.06^ji^
Mean	131.56	123.25	8.30	163.20	39.95	6.68	84.89
SD	64.88	61.95	7.19	69.62	10.89	0.52	2.54
SE	12.98	12.39	1.44	13.92	2.18	0.10	0.51

Abbreviations: RVU, Rapid Viscosity Unit; SD, standard deviation; SE, standard error.

^†^ Results are means of triplicate analysis.

^‡^ Means with same superscripts in the same column are not significant at 5% (*p* < 0.05) level of significance.

### 3.4 Effect of Annealing on Pasting Characteristics of *Elubo*

The results (Figure 5a–c) showed that in both yam species, the fermented yam flour had higher pasting temperature, holding strength, and peak time but lower peak viscosity, breakdown, final viscosity, and setback viscosities compared to the fresh yam paste. During the processing of *elubo*, the yam was blanched in water at 70°C then soaked in water for 24 h before drying and milling. These sequences of processes that the tuber underwent in the processing of “*elubo*” were as a result of physical starch modification. The process is called starch annealing. This is a form of physical starch modification; it involves hydrothermal treatment of starch in excess water at a temperature of > 60°C below the gelatinization temperature of the starch and above glass transition temperature (Yao et al. 2023). Furthermore, the combination of suspension in excess water and heat during *elubo* processing (annealing process) changes the starch structure into a rubbery state that is more mobile, which led to the reorganization of starch amylopectin and amylose into a more compact structure (Anugerah et al. 2022).

From Figure 5b, the pasting temperatures of the “*elubo*” samples were higher than those of their fresh yam pastes. Higher pasting temperature in the *elubo* samples may be adduced to stronger stability of starch structure during annealing due to changes such as leaching of amylose, changes (unraveling of double helices) and increase in the crystalline portion of the annealed starch which made it gelatinize and form paste at a higher temperature compared to native starch. This agrees with the report of Yao et al. (2018), Yao et al. (2023), Yan et al. (2022), Chi et al. (2019) and Jacobs et al. (1995). The Peak Viscosity (PV) of the “*elubo*” samples from both yam species decreased significantly (*p* < 0.05) compared with the PV of the fresh yam paste. The PV of fresh yam paste is higher than that of the “*elubo*” because native starch (in fresh yam paste) is able to absorb more water and gelatinize and become viscous during heating whereas that of the “*elubo*” which has been modified did not gelatinize easily. This is because in annealed starch, the internal structure of the starch is strengthened; this makes it difficult for the starch to imbibe water and hence more energy is required to expand starch chains to trap water molecules. This resulted in a lower swelling capacity of the starches; hence a lower peak viscosity. Lower swelling capacity in starches could also occur as a result of degradation of amylopectin which can lead to unfolding of the double helices and cause decreased granular stability, hence reduced swelling or imbibition of water as a result of ordering rearrangement of starch molecules in the granules because of change of amorphous amylose into a helical form, increase in interactions between amylose chains, and alteration in the interaction between crystallites and the amorphous matrix during annealing (Chen et al. 2017; Eerlingen et al. 1997). Similar results of reduced swelling power were observed for the following annealed starches: Andean oca ( *Oxalis tuberosa* ) starch (Puelles‐Roman et al. 2021), Native sweet potato (Mohamed et al. 2024) fermented cassava (Gomes et al. 2005), unfermented cassava (Gomes et al. 2004), Barley (Waduge et al. 2006), wheat (Lan et al. 2008) and Bambara groundnut (Adebowale and Lawal 2002), Rice (Dias et al. 2010). The effect of annealing on the holding strength viscosity of the starches differs between the two yam species; there was no significant difference (*p* > 0.05) in the holding strength of fresh yam paste in *D. alata* and *D. alata* “*elubo*” whereas the holding strength of *D. rotundata* “*elubo*” was significantly (*p* < 0.05) higher than the holding strength viscosity of fresh *D. rotundata* paste. This could be attributed to the fact that annealing leads to the strengthening of internal structure of the starch, hence more energy is needed to expand starch chains to trap water molecules (Yao et al. 2023). Gomes et al. (2005) reported that annealing leads to the tightening of the amylose‐amylose, amylose‐amylopectin and amylopectin‐amylopectin organization, this crystalline part has been reported to provide dense packing and reinforces the internal structure of the starch which gives it more stability during the hold period and shear stress, hence a higher holding strength. The significantly lower Breakdown viscosity in the *elubo* samples from the two yam species compared to their fresh yam paste may be adduced to their ease of mechanical fragmentation. This is due to the fact that annealing may cause formation of porous surfaces on the starch granule (Yao et al. 2023), which made the granules to be more fragile and prone to erosion and mechanical fragmentation. This may be due to expanding effect phenomenon as a result of excess water in the system, this enabled the granules to absorb excessive amounts of water which in‐turn expands the distance between clusters in the granule and results in pore‐like channels on the surface (Yao et al. 2018). There was no significant difference in the final viscosity (FV) of fresh yam paste and “*elubo*” for *D. rotundata*, though their FV values were higher than that of *D. alata* fresh yam paste and *elubo*. The final viscosity of “*elubo*” from the *D. rotundata* was higher than that of the *D. alata* varieties. This higher final viscosity (thick paste) suggests that the paste of *D. rotundata* was firmer, having greater structural integrity. Previous authors (Yao et al. 2023; Zheng et al. 2023; Zieba et al. 2021; Zhang et al. 2019; Jacobs et al. 1995) have reported that a high final viscosity indicates that annealing made the swollen gelatinized granules or “ghosts” in the paste to be more resistant to mechanical shear; hence formed a more rigid gel during cooling. Setback (SB) viscosity of the fresh yam paste for both yam species was higher than those of the “*elubo*” samples. This may be because they are native starches that have not been modified. Setback viscosity indicates the retrogradation tendency of the starch; hence this implies that the *elubo samples* have lower tendencies to retrograde than the fresh yam paste. Thus, annealing of yam starch is a contributory factor to reducing the extent of retrogradation occurring in yam flour. This is because higher setback viscosity is an indication of re‐association, re‐orientation or retrogradation of starch molecules. This is similar to the observation of Gomes et al. (2004) that reported a reduction in retrogradation in starches due to annealing. It was however observed that the setback of *D. rotundata* “*elubo*” was still higher than that of *D. alata*; hence *D. rotundata elubo* will retrograde more than *D. alata elubo*. This implies it formed a more cohesive paste on cooling compared to those from *D. alata*. This observation was reflected in the textural quality (hardness) of “*amala*” from both yam species. Studies have shown that annealing has an impact on the textural properties of resulting gel products (Mohamed et al. 2024; Zhang et al. 2023). This will have an influence on the textural quality (hardness) of the resulting *amala*.

The peak time of “*elubo*” from both yam species were higher than those from their fresh yam paste (Figure 5c). This may be due to their low swelling power; hence it will take a longer time to reach its peak viscosity compared to samples with higher swelling power.

In general, differences in the pasting characteristics of the *elubo* and fresh yam paste were due to physical reorganization of starch granules, realignment of amylopectin double helices, elevation of gelatinization temperature and reduction of swelling during annealing of the starch in the processing *elubo*. Annealing is a form of physical modification of starch taking place in excess of water (50%–60%) at temperature below the gelatinization point, leading to alteration in the physicochemical properties of starch without destroying its granular structure (Zavareze and Dias 2011). Jayakody and Hoover (2008) reported that annealing changes the physicochemical properties of starches by improving its crystalline perfection and facilitating interactions between starch chains.

It was also observed in this study that annealing had pronounced effect on *D. rotundata* yam starch compared to *D. alata* varieties. This may be as a result of differences in starch structural organization and botanical origin. Otegbayo, Aina, Bokanga, and Asiedu (2005), stated that the histology of raw yams of *D. rotundata* and *D. alata* revealed that starch granules in raw *D. rotundata were* loosely arranged, whereas those in *D. alata* are densely packed. This may be the reason why starches from *D. rotundata* varieties were more annealed than *D. alata* varieties. A greater extent of annealing observed in *elubo* from *D. rotundata* might have influenced the texture of the resulting *amala's* textural quality which were described by the panelists as “soft” and slightly stretchable, unlike *D. alata* that were described as “hard” and slightly stretchable.

**FIGURE 6:**
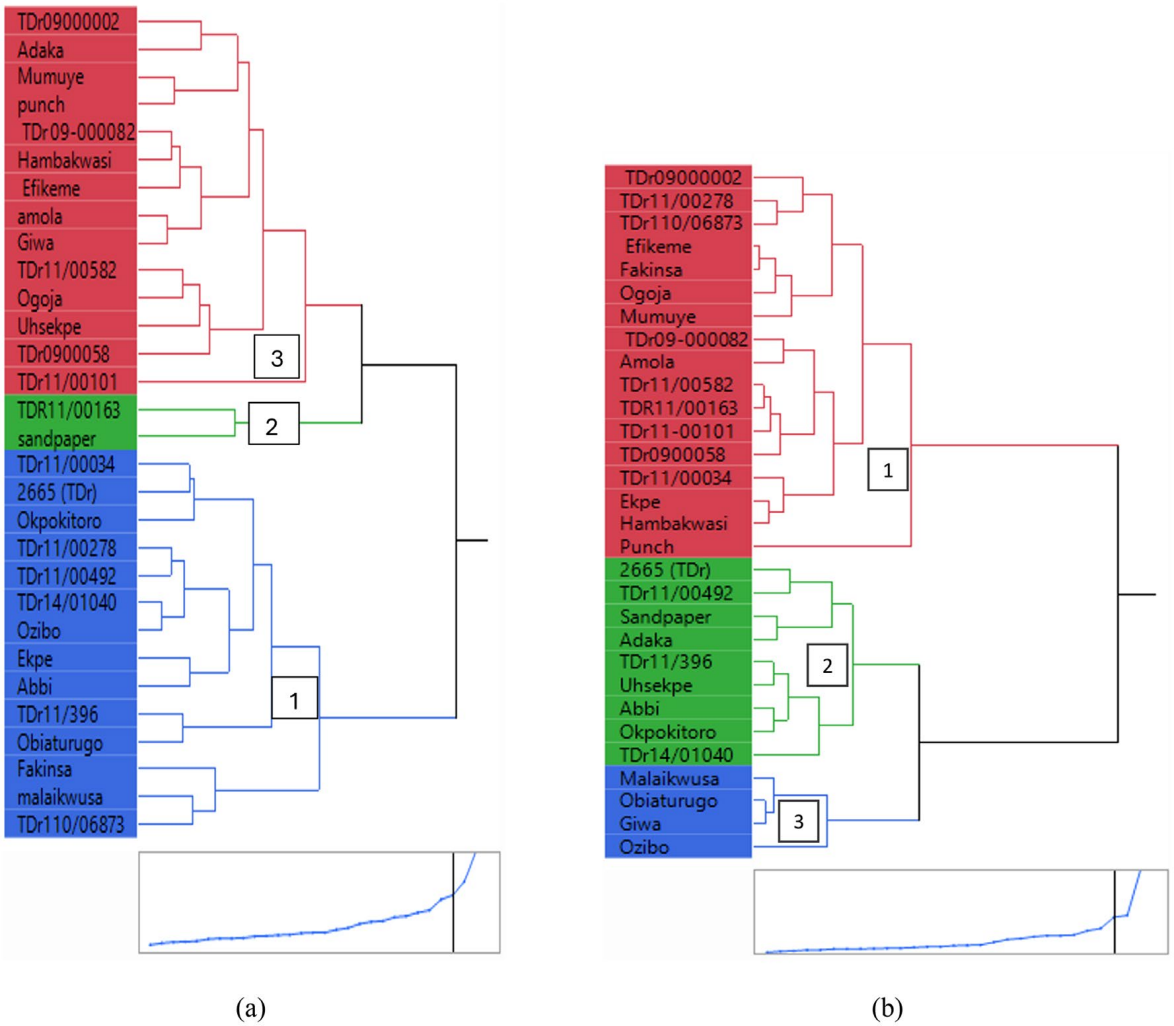
Hierarchical clustering of pasting properties of *Dioscorea rotundata* (a) fresh yam tubers (b) *elubo*.

**FIGURE 7:**
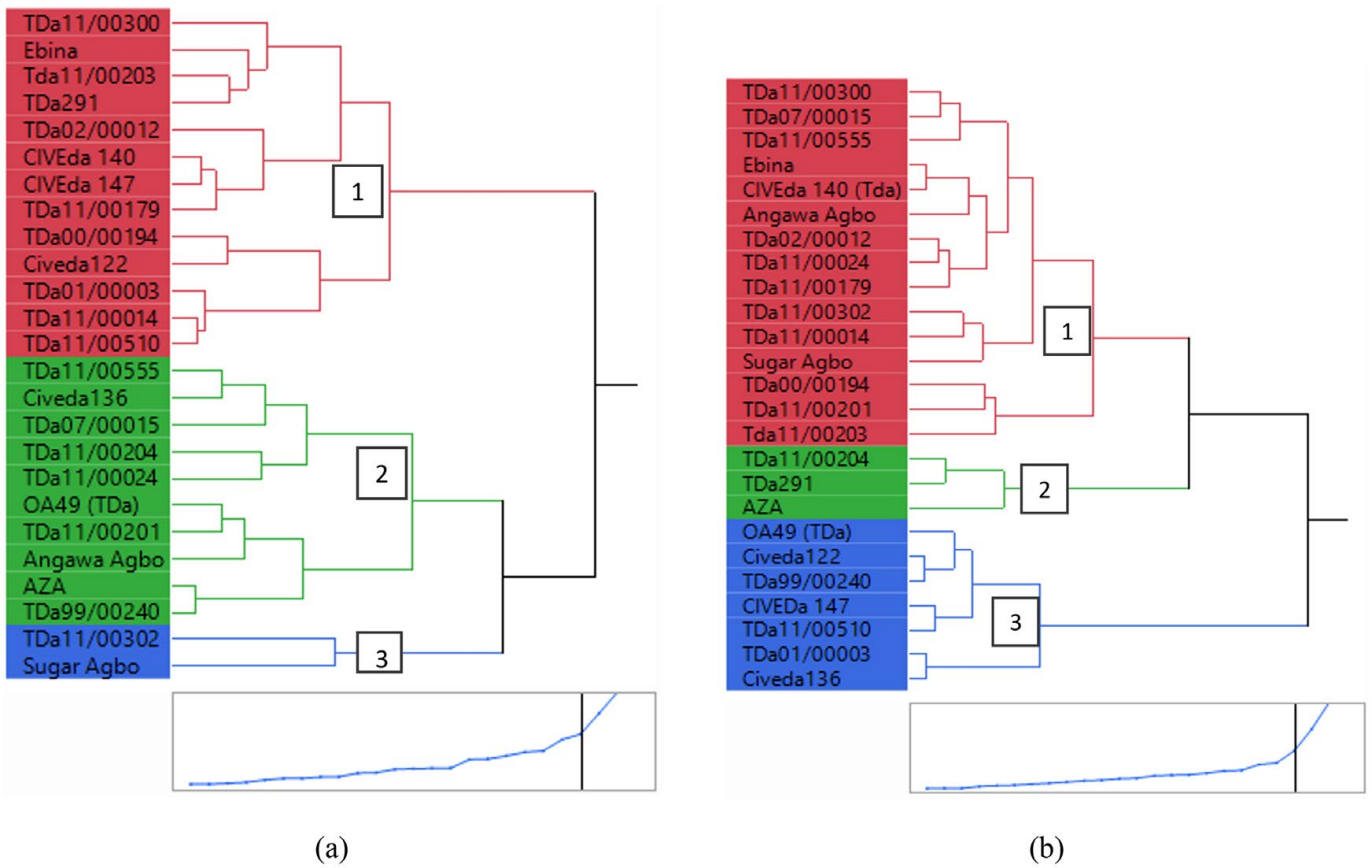
Hierarchical clustering of pasting properties of *Dioscorea alata* (a) fresh yam tubers (b) *elubo*.

The hierarchical clustering of all the pasting properties for *D. rotundata* and *D. alata* fresh tubers and *elubo* samples is presented in Figures 5 and 6, respectively. The two dendrograms in each of the figures show how processing alters pasting behaviors in the same varieties for *D. rotundata* (Figure 6) and *D. alata* (Figure 7). Each cluster (separated by different colors) implies a combination of varieties with similar characteristics in terms of all the pasting properties. Three distinct clusters were observed for each group, with most varieties in cluster one (in red print) being retained after annealing (in *elubo*) for both *D. rotundata* and *D. alata* , implying that the characteristics followed the same trend. Although cluster two (in green) and three (in blue) for both species varied after annealing. Some varieties that cluster together in the fresh paste dendrogram and separated in the fermented flour dendrogram showed that processing altered the relative functional similarity between varieties. The summary of the inferences from this hierarchical clustering is presented in Table 8.

**TABLE 8:** Summary of cluster analysis.

Pasting properties	Cluster 1 (red)	Cluster 2 (green)	Cluster 3 (blue)
Fresh yam of *Dioscorea rotundata*	– Moderate to high peak viscosity (250–400 RVU) – Moderate setback viscosity (95–170 RVU range) – Peak time around 4.5–4.9 min – Pasting temperature mostly in the low–mid 80°C range	– These lower peak viscosity and distinct pasting temperature. They are likely to have different starch composition or granule behavior compared to the other groups	– Higher peak viscosity (some > 500 RVU, e.g., TDr 2665) – Higher breakdown viscosity (> 300 RVU), indicating less stability under heat/shear – Higher setback viscosity (> 200 RVU), meaning stronger retrogradation tendency
*Elubo* of *D. rotundata*	– Moderate to high peak viscosity (260–300 RVU) – Low breakdown values (< 60 RVU), indicating stability under heating/shear – Moderate setback viscosity (80–150 RVU) – Pasting temp often 85°C–90°C	– Peak viscosity can be moderate (260 RVU) – Breakdown viscosity moderate (40–70 RVU) – Pasting temperature in the mid‐high 80 s	– Some have very high peak viscosity (> 300 RVU) – High breakdown (> 90 RVU), indicating lower stability under heating/shear – High setback (> 160 RVU), suggesting stronger gel formation and retrogradation upon cooling
Fresh yam of *Dioscorea alata*	– Mostly moderate‐to‐high peak viscosity (300–450 RVU, some near 450 like Ebina) – Moderate breakdown viscosity (100–265 RVU range) – Moderate setback viscosity (90–180 RVU) – Pasting temperature varies but 70 s to 80°C	– Generally lower to moderate peak viscosity than Cluster 1 – Lower breakdown viscosity, suggesting better stability during heating – Moderate setback viscosity, implying less retrogradation	– They may have distinct starch gelatinization profiles – Could differ sharply in one or more pasting traits (peak, setback, or pasting temp)
*Elubo* of *D. alata*	– Moderate peak viscosity (140–240 RVU) – Low breakdown (< 15 RVU), suggesting high stability under heating/shear – Low setback (40–48 RVU), implying reduced retrogradation tendency – Pasting temperature between 83°C and 90°C	– Very low peak viscosity (55 RVU) – Low to moderate breakdown (5 RVU) – Low setback (42 RVU)	– Extremely low peak viscosity (21 RVU) – Very low holding strength and setback – Low pasting temperature (80°C)

Abbreviation: RVU, Rapid Viscosity Unit.

### 3.5 Relationship Between Pasting Properties of *Elubo* and the Sensory Attributes of *Amala*

**FIGURE 8:**
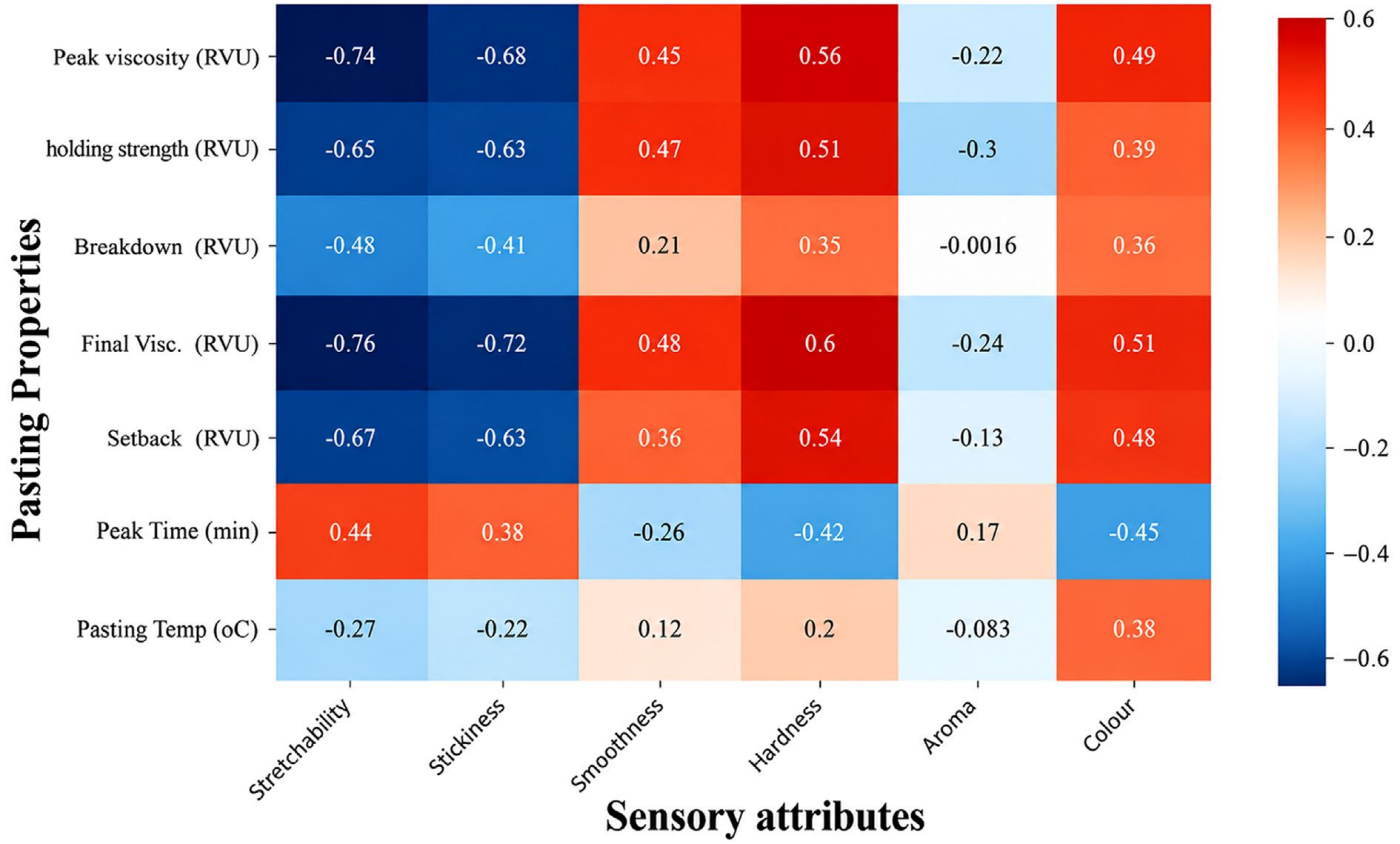
Cross‐correlation heatmap: Pasting properties of *elubo* vs. sensory properties for *Dioscorea rotundata*.

**FIGURE 9:**
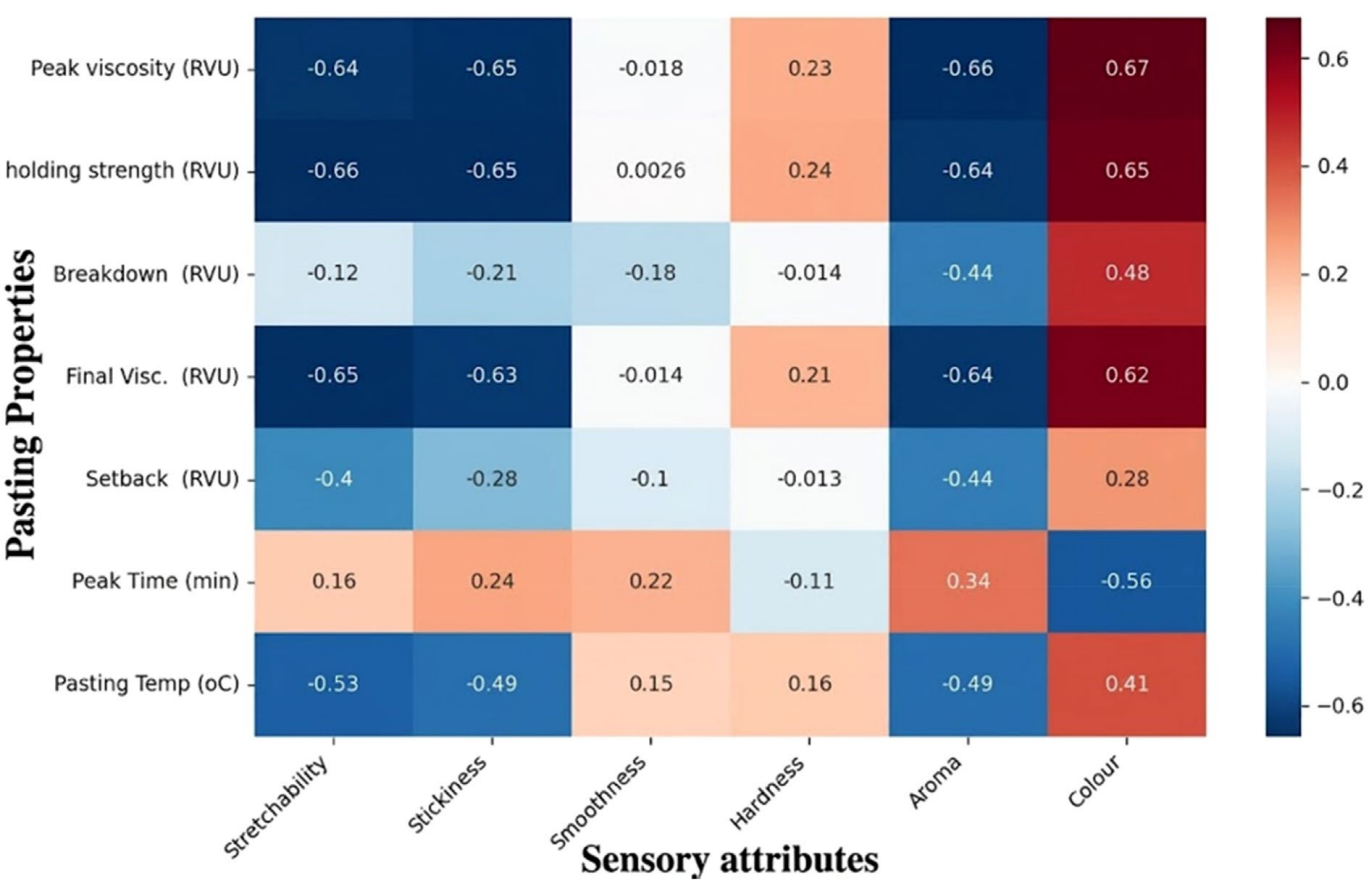
Cross‐correlation heatmap: Pasting properties of *elubo* vs. sensory properties for *Dioscorea alata*.

The correlation analysis presented in Figures 8 and 9 and offers insights into the effects of pasting characteristics of *elubo (*annealed yam flours) on the sensory properties of the derived food product (*amala*) for both *D. rotundata* and *D. alata* , respectively. Generally, pasting characteristics: peak viscosity, holding strength, setback viscosity, and final viscosity of the *elubo* samples were associated with sensory attributes such as stickiness, hardness, stretchability, smoothness, and color of the *amala* produced from them.

For *D. alata* the heatmap showed stronger correlations between pasting properties to stretchability and smoothness which confirmed the high *R* ^2^ (79%) from the regression model (Table 9). Although the heatmap reflects weaker and less consistent correlations among the *D. rotundata* varieties, because some pasting properties relate mildly to sensory traits but with lower overall prediction, which explains why the regression *R* ^2^ was modest (27%) (Table 8); hence the heatmap showed stronger and more consistent correlations between the pasting properties and the sensory results for the *D. alata* varieties. Final viscosity and setback viscosity showed significant correlations with color and hardness of *amala*. These findings suggested that in *D. alata* , higher paste stability and retrogradation influenced the hardness of the *amala*. The strong correlation between setback and hardness has an impact on textural quality of the food product.

**TABLE 9:** Model performance summary.

Species	*R* ^2^	RMSE
*Dioscorea rotundata*	0.2745	0.323
*Dioscorea alata*	0.7882	0.205

For *D. rotundata* , a generally negative correlation was observed between pasting parameters (particularly peak viscosity and final viscosity) and attributes such as stretchability and stickiness. Given the direction of the scoring scale, these negative correlations indicate that increases in viscosity were associated with *amala* samples that were less stretchable and less sticky. These are qualities that are desirable in traditional yam‐based dishes like *amala*. In addition, setback viscosity, a parameter associated with starch retrogradation and reordering of amylose molecules, showed a positive correlation with hardness, suggesting that higher setback values resulted in firmer *amala*. This observation is similar to the report of Kang et al. (2026) and Peroni et al. (2006) that increased setback viscosity leads to tougher gel textures in starch‐rich systems. It is of note that the correlation of the color of the *amala* with the pasting characteristics such as peak viscosity and final viscosity in both yam species could be explained to be due to thermal degradation (non‐enzymatic browning e.g., caramelization, Maillard reactions) that could take place during the heating cycle during pasting characteristics determination.

The contrast in correlation strength between *D. alata* and *D. rotundata* may stem from intrinsic starch differences. These results show that pasting properties, particularly peak viscosity, setback, and final viscosity of fermented yam flour (*elubo*) are significant determinants of the textural quality of *amala*. These can serve as useful information to guide breeders and processors in selecting or modifying yam varieties with optimal properties for consumer‐preferred food products.

## 4 Conclusions

This study showed that annealing of the yam starches, which occurred during the processing of yam fermented flour (*elubo*) (intermediate product), significantly affected its pasting characteristics, which consequently influenced the textural quality of the *amala*, the final product.

The annealed yam flour generally had reduced peak viscosity and setback viscosity, increased pasting temperature, and stability compared to the fresh yam paste. “*Elubo*” from *D. rotundata* had higher peak viscosity, holding strength, breakdown, setback, as well as final viscosity than “*elubo*” from *D. alata* varieties.

From this study, it can be inferred that the effect of starch annealing on yam tubers could be species and varieties dependent as it was observed that starches from the *D. rotundata* species were more “annealed” than those from *D. alata*. This difference could be a result of starch composition (amylose/amylopectin ratio), starch granule arrangement, and botanical origin.

Correlation of pasting properties of the *elubo* (intermediate product) and sensory attributes of their *amala* (final product) showed that pasting characteristics such as peak viscosity, final viscosity, setback viscosity were strongly correlated with sensory attributes such as hardness, stretchability, stickiness and color. This showed that pasting characteristics of *elubo* are significant in determining the textural quality of *amala*. This will be useful in selecting the best varieties for *elubo* with preferred textural quality for *amala* by consumers for optimization of commercial production of *elubo* from either of the two yam species ( *D. alata* and *D. rotundata*).

## Author Contributions

**Olawuyi Yetunde:** investigation, validation, methodology, formal analysis. **Rahman Akinoso:** supervision, validation, visualization, investigation, writing – review and editing. **Bolanle Otegbayo:** conceptualization, investigation, funding acquisition, writing – review and editing, writing – original draft, methodology, visualization, validation, project administration, supervision, resources. **Ayomide Alamu:** methodology, investigation, formal analysis, data curation. **Abiola Tanimola:** investigation, writing – original draft, methodology, visualization, writing – review and editing, software, formal analysis, data curation. **Oluyinka Oroniran:** investigation, validation, project administration, supervision, resources.

## Funding

Bill and Melinda Gates Foundation (grant number: OPP1131414).

## Consent

Verbal and written informed consent was obtained from all study participants; none of the participants were coerced in participating in the study.

## Conflicts of Interest

The authors declare no conflicts of interest.

## Data Availability

The data that support the findings of this study are available from the corresponding author upon reasonable request.
